# Salt Effect Engineering Single Fe‐N_2_P_2_‐Cl Sites on Interlinked Porous Carbon Nanosheets for Superior Oxygen Reduction Reaction and Zn‐Air Batteries

**DOI:** 10.1002/advs.202306599

**Published:** 2024-01-15

**Authors:** Xiaojie Tan, Jinqiang Zhang, Fengliang Cao, Yachao Liu, Hao Yang, Qiang Zhou, Xudong Li, Rui Wang, Zhongtao Li, Han Hu, Qingshan Zhao, Mingbo Wu

**Affiliations:** ^1^ State Key Laboratory of Heavy Oil Processing College of Chemistry and Chemical Engineering College of New Energy China University of Petroleum (East China) Qingdao 266580 China; ^2^ School of Chemical Engineering and Advanced Materials The University of Adelaide Adelaide SA 5005 Australia

**Keywords:** coordination environment, oxygen reduction reaction, salt effect, single‐atom catalyst, Zn‐air battery

## Abstract

Developing efficient metal‐nitrogen‐carbon (M‐N‐C) single‐atom catalysts for oxygen reduction reaction (ORR) is significant for the widespread implementation of Zn‐air batteries, while the synergic design of the matrix microstructure and coordination environment of metal centers remains challenges. Herein, a novel salt effect‐induced strategy is proposed to engineer N and P coordinated atomically dispersed Fe atoms with extra‐axial Cl on interlinked porous carbon nanosheets, achieving a superior single‐atom Fe catalyst (denoted as Fe‐NP‐Cl‐C) for ORR and Zn‐air batteries. The hierarchical porous nanosheet architecture can provide rapid mass/electron transfer channels and facilitate the exposure of active sites. Experiments and density functional theory (DFT) calculations reveal the distinctive Fe‐N_2_P_2_‐Cl active sites afford significantly reduced energy barriers and promoted reaction kinetics for ORR. Consequently, the Fe‐NP‐Cl‐C catalyst exhibits distinguished ORR performance with a half‐wave potential (E_1/2_) of 0.92 V and excellent stability. Remarkably, the assembled Zn‐air battery based on Fe‐NP‐Cl‐C delivers an extremely high peak power density of 260 mW cm^−2^ and a large specific capacity of 812 mA h g^−1^, outperforming the commercial Pt/C and most reported congeneric catalysts. This study offers a new perspective on structural optimization and coordination engineering of single‐atom catalysts for efficient oxygen electrocatalysis and energy conversion devices.

## Graphical abstract

A novel single‐atom electrocatalyst with superior Fe‐N_2_P_2_‐Cl active sites is successfully synthesized through a salt effect‐induced strategy. The salt effect facilitates the formation of Fe‐N_2_P_2_‐Cl configuration, achieving significantly reduced energy barriers and promoting ORR kinetics. The induced interlinked nanosheet microstructure with hierarchical pores promotes the exposure of Fe‐N_2_P_2_‐Cl and provides rapid mass/electron transfer channels, guaranteeing the outstanding electrocatalytic performance of Zn‐air battery devices.

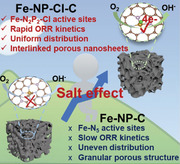

## 1 Introduction

The advancement of renewable energy sources and energy conversion technologies is crucial to addressing energy consumption and environmental concerns. Zn‐air battery has gained widespread attention due to its high energy density, zero emissions, safety, and reliability.^[^ ^1^ ^]^ However, its large‐scale application is limited by the inferior activity and sluggish kinetics of oxygen reduction reaction (ORR) at the air cathode.^[^ ^2^ ^]^ Although platinum‐based electrocatalysts (e.g., Pt/C) have demonstrated excellent performance in ORR, the scarcity and high cost unfortunately hinder their upscaling.^[^ ^3^ ^]^ Therefore, non‐noble metal catalysts with efficient oxygen electrocatalytic activity and satisfactory durability are highly desired as replacements for Pt‐based electrocatalysts.^[^ ^4^ ^]^

Non‐noble metal‐nitrogen‐carbon (M‐N‐C) catalysts, especially Fe‐N‐C single‐atom catalysts, are regarded as one of the most promising substitutes due to their favorable ORR activities, maximum atom‐utilization efficiency, and customizable electronic structures.^[^ ^5^ ^]^ Multiple Fe‐N‐C catalysts with atomic Fe‐N_x_ sites have been reported as efficient promoters for ORR and Zn‐air batteries.^[^ ^6^ ^]^ Recent studies have demonstrated that modifying the metal‐centered coordination by heteroatom doping (such as S, O, and P, etc.) can effectively tailor the electronic structure of Fe‐N‐C catalysts and optimize the adsorption of intermediates, thereby regulating the intrinsic activity.^[^ ^7^ ^]^ For instance, Li et al. synthesized an asymmetric N, S‐coordinated single‐atom Fe catalyst with extra‐axial fifth OH coordination (Fe‐N_3_S_1_OH) on N, S co‐doped porous carbon nanospheres (Fe‐N/S‐C), presenting a superior ORR performance with a half‐wave potential (E_1/2_) of 0.882 V and high peak power density of 203 mW cm^−2^ for Zn‐air battery.^[^ ^8^ ^]^ Zhou et al. incorporated P atoms into the second coordination sphere to construct a P/Fe‐N‐C single‐atom catalyst, which resulted in a local distortion around Fe and balanced the *OOH/*O adsorption at Fe‐N_4_ sites to achieve an enhanced E_1/2_ of 0.90 V for ORR, confirming the validity of dependence of atomic metal coordination on the catalytic performance.^[^ ^9^ ^]^

Additionally, considering the three‐phase reaction interfaces on the air cathode, characteristics of the carbon matrix that dramatically affect the diffusion and adsorption of O_2_ molecules and electron transmission also play an essential role in the ORR and Zn‐air battery performances.^[^ ^10^ ^]^ Generally, a hierarchical porous structure is valid to facilitate mass transfer and exposure of the active sites in the ORR process.^[^ ^11^ ^]^ For example, Tang et al. prepared a hierarchical meso‐/microporous N/S co‐doped carbon nanocage with atomic Fe‐N_4_ sites through a nano‐emulsion‐induced polymerization self‐assembly method from Zn‐based zeolitic imidazolate framework‐8 (ZIF‐8), which exhibited an excellent ORR performance with an E_1/2_ of 0.91 V.^[^ ^12^ ^]^ However, most reported porous catalysts, especially ZIF‐derived catalysts, are generally constructed with micropores and mesopores ≈2–4 nm.^[^ ^13^ ^]^ The narrowly distributed micropores may impede the rapid permeation of electrolyte and reactant diffusion, resulting in limited mass transfer of the oxygen reaction‐related species.^[^ ^14^ ^]^ Therefore, apart from rational engineering of the coordination environment, constructing specific carbon matrices with accessible mass transport channels is of equal significance to realize the high intrinsic activity of atomic metal centers. A feasible approach to synergistically tailor the matrix microstructure and coordination environment of M‐N_x_ active sites for advanced M‐N‐C electrocatalysts is still immensely desirable.

Herein, we demonstrate a novel salt effect‐induced strategy to fabricate an advanced single‐atom Fe catalyst (Fe‐NP‐Cl‐C) for superior ORR and Zn‐air batteries. The existence of NaCl triggers a conspicuous salt effect to promote the ionization equilibrium of phytic acid (PA) and enhances its chelation interaction with Fe ions, followed by assembling with o‐phenylenediamine (OPD). After carbonization and acid etching, N and P coordinated atomically dispersed Fe with extra‐axial Cl is engineered on a matrix of interlinked carbon nanosheets with hierarchical pores. In this process, the salt effect not only facilitates the formation of single Fe‐N_2_P_2_‐Cl active centers but also induces the interlinked nanosheet architecture with hierarchical pores. Thanks to the favorable matrix structure and coordination environment, the resulting Fe‐NP‐Cl‐C catalyst exhibits outstanding ORR performance with an E_1/2_ of 0.92 V and excellent stability. Interestingly, the Zn‐air battery based on Fe‐NP‐Cl‐C demonstrates an open‐circuit voltage of 1.495 V, an extremely high peak power density of 260 mW cm^−2^, and a large specific capacity of 812 mA h g^−1^, far exceeding the commercial Pt/C and Fe‐NP‐C single‐atom reference catalyst obtained without NaCl salt.

## 2 Results and Discussion

### 2.1 Synthesis and Characterization of Catalysts

**Figure 1:**
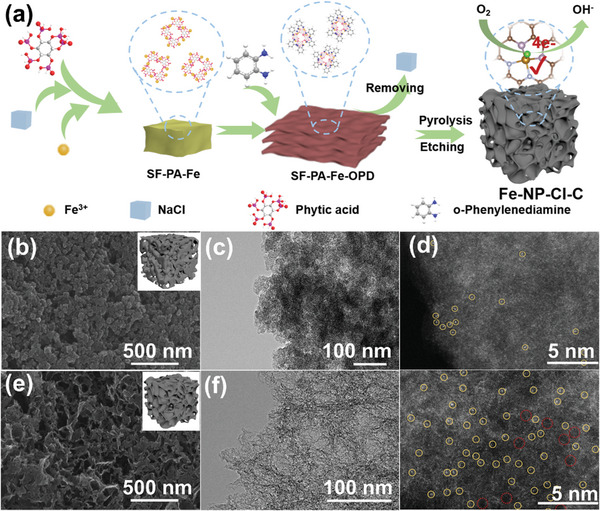
a) Schematic illustration for the synthesis of Fe‐NP‐Cl‐C. b) SEM image and schematic structure (inset) of Fe‐NP‐C. c) TEM image and d) HAADF‐STEM image of Fe‐NP‐C. Fe sites are marked by yellow circles. e) SEM image and schematic structure (inset) of Fe‐NP‐Cl‐C. f) TEM image and g) HAADF‐STEM image of Fe‐NP‐Cl‐C. Atomic Fe sites are marked with yellow circles, and the micropores on the matrix are marked with red circles.

The preparation procedure of Fe‐NP‐Cl‐C is shown in **Figure** **1**a. NaCl salt was dissolved and dispersed in PA solution, followed by chelation with Fe precursor (Fe^3+^) to obtain SE‐PA‐Fe. The presence of Na^+^ and Cl^−^ favors the ionization of PA induced by the increased ion concentration and intensified electrostatic interaction, thereby provoking stronger chelation with Fe ions to achieve their homogeneous immobilization (Scheme S1, Supporting Information). Notably, the introduction of Cl^−^ occupies partial phosphate groups of PA, which would further isolate the anchored Fe ions and facilitate the formation of atomically dispersed metal centers. Subsequently, OPD was added and assembled with the uniformly dispersed SE‐PA‐Fe to achieve the SE‐PA‐Fe‐OPD intermediate. After removing the NaCl salt through water washing, N and P coordinated atomically dispersed Fe with extra‐axial Cl was synthesized on interlinked porous carbon nanosheets through carbonization and acid etching. For comparison, Fe‐NP‐C and Fe‐N‐Cl‐C were prepared with the same method except for the addition of NaCl or PA, respectively, whereas Fe‐N‐C was fabricated without the inclusion of NaCl and PA. To distinguish the influence of salt, various congeners including Fe‐NP‐Cl‐C‐LiCl, Fe‐NP‐F‐C‐NaF, Fe‐NP‐C‐NaNO_3_, Fe‐NP‐Br‐C‐NaBr, Fe‐NP‐Cl‐C‐KCl, and Fe‐NP‐Cl‐C‐MgCl_2_ were prepared by substituting LiCl, NaF, NaNO_3_, NaBr, KCl, and MgCl_2_ for NaCl, respectively. Fe‐NP‐Cl‐C‐ST was synthesized by preserving NaCl as a salt template rather than removing it.

As shown in Figure S1 (Supporting Information), the visual color of PA‐Fe and SE‐PA‐Fe‐NaNO_3_ appears yellow, which intensifies into luminous yellow upon the introduction of chloride salts (i.e., NaCl, KCl, and MgCl_2_), while changing brown with the addition of NaBr. This phenomenon suggests an obvious impact on the nanostructure of PA‐Fe. pH detection further confirms the promoted ionization equilibrium of PA in the presence of various salts, leading to lower pH values varying from 1.15 to −0.07 (Table S1, Supporting Information). The salting‐out ability of salt intensifies as the cation size decreases, with a notable exception of Li^+^ which is as weak as Cs^+^. As to the anion, the strong affinity toward Fe^3+^ impairs the influence of F^−^. Considering these factors, NaCl demonstrates the most conspicuous salt effect.^[^ ^15^ ^]^ Scanning electron microscopy (SEM) images show a smooth bulk structure of PA‐Fe, and PA‐Fe‐OPD exhibits a rough surface with a length ranging from 40 to 100 µm (Figure S2, Supporting Information). In contrast, the introduction of salt leads to a coarse bulk structure of SE‐PA‐Fe, which presents decreased size and more wrinkles after assembling with OPD (SE‐PA‐Fe‐OPD). Fourier transform infrared spectroscopy (FT‐IR) spectra of PA‐Fe and SE‐PA‐Fe exhibit prominent peaks at 1080 and 1637 cm^−1^, respectively, corresponding to PO_4_^3−^ and HPO_4_^2−^ originating from PA (Figure S3, Supporting Information).^[^ ^16^ ^]^ Meanwhile, the peak at 1533 cm^−1^ in PA‐Fe‐OPD and SE‐PA‐Fe‐OPD can be ascribed to C = N vibration, indicating the chemical crosslinking between PA and OPD.^[^ ^17^ ^]^ In line with the inductively coupled plasma‐atomic emission spectroscopy (ICP) testing results listed in Table S2 (Supporting Information), no diffraction peak of NaCl can be identified from the X‐ray diffraction (XRD) patterns of the samples, implying complete removal of the NaCl salt from SE‐PA‐Fe and SE‐PA‐Fe‐OPD before carbonization.

SEM and transmission electron microscopy (TEM) images were employed to illustrate the morphology of Fe‐NP‐C and Fe‐NP‐Cl‐C. In the absence of NaCl salt, Fe‐NP‐C shows a granular and porous arrangement, with carbon stacking observed in the TEM images (Figure 1b,c; Figure S4, Supporting Information). In contrast, both Fe‐NP‐Cl‐C (Figure 1e,f; Figure S5, Supporting Information) and Fe‐NP‐C‐NaNO_3_ (Figure S6, Supporting Information) present an interconnected porous framework composed of lamellar nanosheets, illustrating that the microstructure of the carbon matrix can be modulated by salt effect.^[^ ^14^, ^18^ ^]^ High‐resolution transmission electron microscopy (HRTEM) images of Fe‐NP‐C and Fe‐NP‐Cl‐C confirm the absence of Fe nanoclusters or nanoparticles in the samples (Figures S4 and S5, Supporting Information). High‐angle annular dark‐field‐scanning transmission electron microscope (HAADF‐STEM) image of Fe‐NP‐C exhibits inhomogeneous bright dots marked by yellow circles corresponding to single Fe atoms (Figure 1d). This should be attributed to the uneven dispersion of Fe precursor, thereby resulting in metal agglomeration during carbonization. Whereas Fe‐NP‐Cl‐C shows abundant highly dispersed and isolated bright dots, indicating that atomic Fe sites are uniformly distributed on the matrix (Figure 1g). As discussed above, the salt effect can trigger isolated and robust immobilization of Fe precursor, which realizes homogeneous accommodation of atomically dispersed Fe centers. Compared with Fe‐NP‐C, the dark space marked with red circles identifies the presence of more micropores in Fe‐NP‐Cl‐C. Element distribution was evaluated by energy dispersive X‐ray (EDX) mapping, which manifests a homogeneous distribution of C, N, P, and Fe elements in Fe‐NP‐C, with additional Cl signal in Fe‐NP‐Cl‐C (Figures S7 and S8, Supporting Information). Moreover, Fe‐NP‐C and Fe‐NP‐Cl‐C deliver air‐water contact angles of 118^o^ and 134^o^, respectively, demonstrating the heightened hydrophobicity of Fe‐NP‐Cl‐C attributed to the intercalation of Cl (Figure S9, Supporting Information). It is noteworthy that Fe‐NP‐Cl‐C manifests a significantly reduced contact angle of 50° for an O_2_‐saturated KOH solution, which is even lower than that of Fe‐NP‐C (64^o^), suggesting that oxygen is more favorably released from the electrolyte and diffuses through the hydrophobic Fe‐NP‐Cl‐C electrode. Such hydrophobic nature would engender abundant solid‐liquid‐gas three‐phase reaction interfaces and facilitate oxygen diffusion on the air cathode of the Zn‐air battery.^[^ ^19^ ^]^

XRD patterns of Fe‐NP‐C and Fe‐NP‐Cl‐C show two broad peaks corresponding to the (002) and (100) diffractions of graphitic materials (Figure S10, Supporting Information). Potentially due to the introduction of axial Cl atoms, the (002) peak of Fe‐NP‐Cl‐C shows a slight shift to a lower diffraction angle in comparison to Fe‐NP‐C, resulting in an enlarged lattice spacing of the carbon layer.^[^ ^20^ ^]^ Both Fe‐NP‐C and Fe‐NP‐Cl‐C present peaks related to defective carbon (D band) and graphitic carbon (G band) at 1350 and 1590 cm^−1^ in the Raman spectra, with *I_D_*/*I_G_* ratios of 0.93 and 1.12, respectively. Compared with Fe‐NP‐C, Fe‐NP‐C‐NaNO_3_, Fe‐NP‐Br‐C‐NaBr, and Fe‐NP‐Cl‐C‐KCl also show increased *I_D_*/*I_G_* ratios of 0.96, 0.95, and 0.96 in Figure S11 (Supporting Information), indicating that salt effect induces the generation of more defects. This should be attributed to the promoted ionization of PA in the presence of salts, which results in the formation of SE‐PA‐Fe‐OPD precursor with smaller size and more structural defects. N_2_ adsorption/desorption measurements reveal that Fe‐NP‐Cl‐C affords a specific surface area of 783 m^2^ g^−1^, which is larger than that of Fe‐NP‐C (528 m^2^ g^−1^). Corresponding pore size distribution shows the coexistence of micropores and mesopores in Fe‐NP‐C and Fe‐NP‐Cl‐C. Notably, in contrast to the mesopores primarily distributed at ≈30 nm for Fe‐NP‐C, Fe‐NP‐Cl‐C mainly possesses micropores and mesopores within a range below 10 nm. This phenomenon validates the hierarchical porous nanosheet structure of Fe‐NP‐Cl‐C induced by the salt effect, which is beneficial to the diffusion of reactants and exposure of active sites.

**Figure 2:**
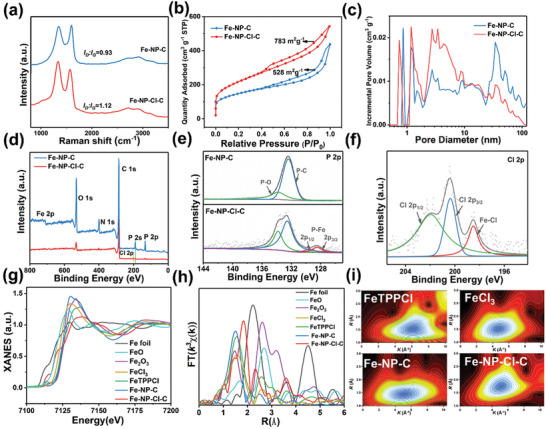
a) Raman spectra of Fe‐NP‐C and Fe‐NP‐Cl‐C. b) N_2_ adsorption/desorption isotherms and c) corresponding pore size distribution of Fe‐NP‐C and Fe‐NP‐Cl‐C. d) XPS survey spectra of Fe‐NP‐C and Fe‐NP‐Cl‐C. e) P 2p XPS spectra of Fe‐NP‐C and Fe‐NP‐Cl‐C. f) Cl 2p XPS spectrum of Fe‐NP‐Cl‐C. g) Fe K‐edge XANES spectra and (h) *k^3^*‐weighted FT spectra in R space for Fe‐NP‐C and Fe‐NP‐Cl‐C, and the data of Fe foil, FeO, Fe_2_O_3_, FeCl_3_, FeTPPCl are shown as references. (i) Wavelet transforms of Fe K‐edge EXAFS for FeTPPCl, FeCl_3_, Fe‐NP‐C, and Fe‐NP‐Cl‐C.

X‐ray photoelectron spectroscopy (XPS) survey spectra in **Figure** **2**d indicate the presence of C, N, O, P, and Fe in Fe‐NP‐C and Fe‐NP‐Cl‐C, and an additional Cl signal can be observed in the spectrum of Fe‐NP‐Cl‐C, confirming the successful accommodation of Cl atoms into the carbon matrix. The C 1s XPS spectra exhibit four peaks at 284.7, 285.6, 286.7, and 288.8 eV, corresponding to C = C/C‐C, C‐N, C‐O, and O‐C = O bonds, respectively (Figure S12a,b, Supporting Information). The N 1s spectra (Figure S12c, Supporting Information) can be deconvoluted into pyridinic N (398.5 eV), Fe‐N_x_ (399.4 eV), pyrrolic N (399.8 eV), graphitic N (401.4 eV), and N‐O (404.0 eV). It can be found that Fe‐NP‐Cl‐C shows a higher percentage of pyridinic and graphitic N compared to Fe‐NP‐C, which would be conducive to the proceeding of ORR.^[^ ^21^ ^]^ In terms of the P 2p spectra shown in Figure 2e, both Fe‐NP‐C and Fe‐NP‐Cl‐C exhibit peaks of P‐C (132.5 eV) and P‐O (133.8 eV), with additional Fe‐P peaks (128.5 and 129.5 eV)^[^ ^22^ ^]^ in Fe‐NP‐Cl‐C. The Fe‐P peak can also be detected in Fe‐NP‐C‐NaNO_3_, Fe‐NP‐Br‐C‐NaBr, and Fe‐NP‐Cl‐C‐KCl (Figure S13, Supporting Information), Importantly, although Fe‐NP‐C shows a much higher content of P than Fe‐NP‐Cl‐C, the P atoms are mainly doped in the carbon matrix rather than coordinated with the Fe centers (Tables S3 and S4, Supporting Information). The Cl 2p spectrum of Fe‐NP‐Cl‐C displays peaks centered at 198.5, 200.4, and 201.9 eV, which are ascribed to Fe‐Cl, Cl 2p_3/2_, and Cl 2p_1/2_, respectively (Figure 2f).^[^ ^23^ ^]^ Interestingly, Fe‐Cl and Fe‐Br bonds^[^ ^24^ ^]^ can also be observed in Fe‐NP‐Cl‐C‐KCl and Fe‐NP‐Br‐C‐NaBr, respectively (Figure S14, Supporting Information). Accordingly, it is evident that the salt effect efficiently facilitates the formation of Fe‐P and Fe‐halogen bonds, which can controllably modulate the coordination configuration of atomic Fe centers.

X‐ray absorption near edge structure (XANES) and extended X‐ray absorption fine structure (EXAFS) measurements were conducted to provide a deeper understanding of the coordination and local chemical environment of the single‐atom catalysts. Figure 2g shows the Fe K‐edge XANES of Fe‐NP‐C, Fe‐NP‐Cl‐C, Fe foil, FeO, Fe_2_O_3_, FeCl_3_, and FeTPPCl, respectively. The absorption edges of Fe‐NP‐C and Fe‐NP‐Cl‐C are located between that of FeO and Fe_2_O_3_, suggesting the oxidation stages of Fe are both between +2 and +3. By comparison with Fe‐NP‐C, the valence of Fe in Fe‐NP‐Cl‐C is closer to FeO (+2), revealing the less positively charged Fe species arising from the simultaneous incorporation of P and Cl.^[^ ^25^ ^]^ Fourier‐transformed (FT) *k^3^*‐weighted EXAFS curves of Fe‐NP‐C and Fe‐NP‐Cl‐C reveal the presence of a Fe‐N coordination shell at R space of 1.5 Å (Figure 2h). Remarkably, a broad and dominated peak at 1.8 Å can be observed in Fe‐NP‐Cl‐C, corresponding to the scatterings of Fe‐P (1.9 Å) and Fe‐Cl (1.7 Å). It is worth mentioning that the relatively weak peak at 2.35 Å can be attributed to the second Fe‐C shell contribution, which is inconsistent with the Fe‐Fe signal at 2.18 Å from Fe foil (Figure S15a, Supporting Information), indicating no metallic Fe cluster or nanocrystal.^[^ ^22^, ^26^ ^]^ Furthermore, quantitative least‐squares EXAFS curve‐fitting analysis was carried out to further elucidate the structural parameters of Fe sites. According to the fitting results for Fe‐NP‐C, one Fe atom is coordinated to five first‐shell N atoms (Fe‐N_5_) with an average bond length of 2.02 Å (Figure S16a,b, Supporting Information). Compared to FeTPPCl, the slight shift of Fe‐N suggests that P is located at the outer shell of the Fe‐N_5_ center and affects the electronic state of Fe (Figure S15b, Supporting Information). In contrast, fitting of Fe‐NP‐Cl‐C exhibits an average coordination number of 2.3 for Fe‐N at 2.07 Å, 2.0 for Fe‐P at 2.4 Å and 0.6 for axial Fe‐Cl at 2.2 Å, which can be confirmed as Fe‐N_2_P_2_‐Cl configuration (Figure S16c,d and Table S6, Supporting Information). Wavelet transform intensities of Fe‐NP‐C and Fe‐NP‐Cl‐C are clearly distinguished from Fe‐Fe, Fe‐O, or Fe‐Cl paths in Fe foil, Fe_2_O_3_, and FeCl_3_ (Figure 2i, Figure S16e,f, Supporting Information). By comparison with Fe‐NP‐C, Fe‐NP‐Cl‐C displays an obvious migration due to the existence of Fe‐P and Fe‐Cl coordination.^[^ ^27^ ^]^ These findings demonstrate a salt effect is an effective approach for engineering the coordination environment of the Fe single‐atom catalyst, delivering N, P coordinated Fe atoms with extra‐axial Cl coordination on the carbon matrix.

### 2.2 Electrocatalytic Performance

**Figure 3:**
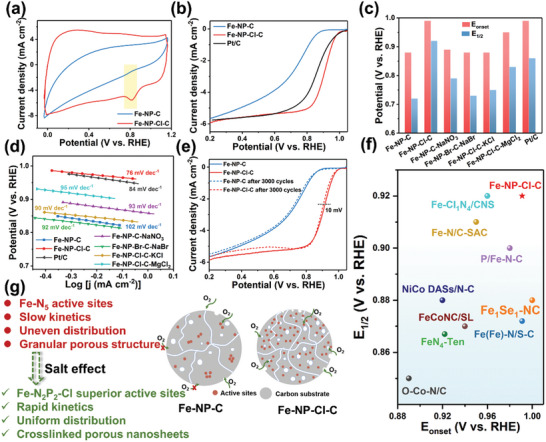
a) CV curves of Fe‐NP‐C and Fe‐NP‐Cl‐C in O_2_‐saturated 0.1 M KOH solution. b) LSV curves of Fe‐NP‐C, Fe‐NP‐Cl‐C, and commercial Pt/C (20 wt.%) in O_2_‐saturated 0.1 M KOH solution at a rotating speed of 1600 rpm. c) E_onset_ and E_1/2_ for the various catalysts. d) Tafel plots of the catalysts derived from mass transport correction of corresponding LSV data. (e) LSV curves of Fe‐NP‐C, Fe‐NP‐Cl‐C before and after 3000 cycles. f) Comparison of the E_onset_ and E_1/2_ for Fe‐NP‐Cl‐C and reported catalysts listed in Table S7 (Supporting Information). g) Schematic illustration of salt effect on the physicochemical properties of the catalysts.

The electrocatalytic performance of the catalysts was evaluated using a rotating disk electrode (RDE) and rotating ring disk electrode (RRDE) in an O_2_‐saturated 0.1 M KOH solution. The cyclic voltammetry (CV) curves indicate that Fe‐NP‐Cl‐C has a more pronounced reduction peak compared to Fe‐NP‐C (**Figure** **3**a). The linear sweep voltammetry (LSV) curves at 1600 rpm shown in Figure 3b and Figure S17 (Supporting Information) reveal that the onset potential (E_onset_) of Fe‐NP‐Cl‐C is ≈0.99 V, which is comparable to the commercial Pt/C catalyst. Notably, the E_1/2_ of Fe‐NP‐Cl‐C is measured to be 0.92 V, significantly higher than that of Fe‐N‐Cl‐C (0.76 V), Fe‐NP‐C (0.72 V), Fe‐N‐C (0.69 V), and commercial Pt/C (0.86 V), demonstrating the superior intrinsic ORR activity of Fe‐NP‐Cl‐C. It is worth mentioning that Fe‐NP‐Cl‐C‐ST shows a much inferior ORR performance than Fe‐NP‐Cl‐C, which may be attributed to the collapse of interlinked porous architecture arising from the excessive salt template. Moreover, the ORR activity of Fe‐NP‐Cl‐C is affected by the NaCl salt dosage, which rises first and then falls with increasing NaCl concentration, and all samples exhibit better ORR performance than that of Fe‐NP‐C (Figure S19, Supporting Information). A summary of E_onset_ and E_1/2_ for the various catalysts unveils the introduction of different salts that make significant contributions to the promotion of catalytic properties (Figure 3c; Figures S20 and S21, Supporting Information). As shown in Figure 3d, the Tafel slope of Fe‐NP‐Cl‐C is smaller than those of Pt/C and other reference samples, manifesting reinforced ORR kinetics over Fe‐NP‐Cl‐C. The OER test of the samples was further investigated and shown in Figure S22 (Supporting Information). Fe‐NP‐Cl‐C possesses an OER potential of 1.69 V at 10 mA cm^−2^, apparently lower than that of Fe‐NP‐C (1.93 V). In addition, the presence of salt effect results in promoted OER performances for Fe‐NP‐C‐NaNO_3_ (1.81 V), Fe‐NP‐Br‐C‐NaBr (1.89 V), Fe‐NP‐Cl‐C‐KCl (1.88 V), and Fe‐NP‐Cl‐C‐MgCl_2_ (1.75 V). Apart from Li^+^ and F^−^, the salting‐out ability increases with the decreasing of cation size, following the order of K^+^ > Mg^2+^ > Na^+^ and Br^−^ > Cl^−^. Therefore, NaCl stands out among the various salts due to its suitable ionic size. Based on the aforementioned characterizations and electrochemical measurements, the impressive performance of Fe‐NP‐Cl‐C can be attributed to the following reasons, as depicted in Figure 3g. The salt effect of NaCl facilitates the formation and uniform distribution of atomic Fe sites with a unique Fe‐N_2_P_2_‐Cl configuration to achieve rapid ORR kinetics. Meanwhile, the interlinked carbon nanosheet matrix with abundant hierarchical pores facilitates the exposure of Fe‐N_2_P_2_‐Cl active sites and provides efficient mass/electron transfer pathways, guaranteeing smooth transformation of the ORR process.

The rotating ring‐disk electrode experiments were conducted to investigate the ORR pathway over Fe‐NP‐C and Fe‐NP‐Cl‐C. As can be seen in Figure S23 (Supporting Information), Fe‐NP‐Cl‐C shows a much lower H_2_O_2_ yield than Fe‐NP‐C within the potential range of 0.45 to 0.6 V, and the average electron transfer number is determined to be 3.95, implying a four‐electron pathway on Fe‐NP‐Cl‐C. Furthermore, the exceptional stability of Fe‐NP‐Cl‐C is demonstrated by a slight negative shift of E_1/2_ by 10 mV after 3000 cycles (Figure 3e). This is further verified by i‐t measurements, revealing a relative current of 91% even after 18 h for Fe‐NP‐Cl‐C, whereas the commercial Pt/C presents a rapid fading to 68% within 12 h (Figure S24, Supporting Information). Remarkably, both Fe‐NP‐C and Fe‐NP‐Cl‐C exhibit excellent methanol tolerance ability, in contrast to Pt/C which experiences a conspicuous decrease in current density upon methanol injection (Figure S25, Supporting Information). To further clarify the dominant role of single‐atom Fe‐related structure in ORR for Fe‐NP‐C and Fe‐NP‐Cl‐C, a KSCN poisoning experiment was conducted.^[^ ^28^ ^]^ As shown in Figure S26 (Supporting Information), the ORR activity of both Fe‐NP‐C and Fe‐NP‐Cl‐C dramatically diminishes after adding KSCN, indicating a significant contribution of the atomic Fe‐N_5_ for Fe‐NP‐C and atomic Fe‐N_2_P_2_‐Cl for Fe‐NP‐Cl‐C, respectively. Gratifyingly, the single‐atom Fe‐NP‐Cl‐C catalyst shows prominent ORR performance compared to most recently reported Fe/Co‐based ORR electrocatalysts (Figure 3f; Table S7, Supporting Information).

**Figure 4:**
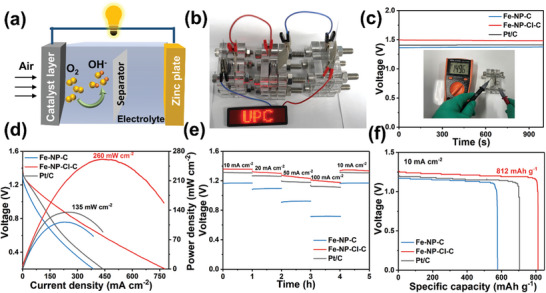
a) Graphical illustration for the Zn‐air battery device. b) Photograph of red LED screen powered by three Fe‐NP‐Cl‐C‐based Zn‐air batteries in series. c) Initial open‐circuit potential and (d) polarization curves and corresponding power density plots of Zn‐air batteries with Fe‐NP‐C, Fe‐NP‐Cl‐C, and Pt/C as the air cathodes. e) Discharge profiles of the Zn‐air batteries at current densities of 10, 20, 50, 100, and 10 mA cm^−2^. f) Long‐time galvanostatic discharge curves at 10 mA cm^−2^ for the assembled Zn‐air batteries.

Motivated by the satisfactory ORR performance, Fe‐NP‐Cl‐C was further employed as an air cathode for Zn‐air batteries. The device arrangement is depicted in **Figure** **4**a. For comparison, the Fe‐NP‐Cl‐C and Pt/C electrodes were also tested under the same conditions. As illustrated in Figure 4b and Figure S27 (Supporting Information), three Fe‐NP‐Cl‐C‐based batteries connected in series are capable of powering a lighting‐emitting diode (LED) screen and an electric fan, indicating its great potential as a cathode material. The initial open‐circuit voltage (Figure 4c) of Fe‐NP‐Cl‐C‐based Zn‐air battery is determined to be 1.495 V, which distinctly surpasses that of Fe‐NP‐C and Pt/C. Notably, the Fe‐NP‐Cl‐C‐based battery also displays a significantly higher peak power density of 260 mW cm^−2^ than the other two batteries based on Fe‐NP‐C (110 mW cm^−2^) and Pt/C (135 mW cm^−2^) as depicted in Figure 4d. Additionally, the charge‐discharge curves indicate that Fe‐NP‐Cl‐C possesses reliable rechargeability and electrocatalytic activities (Figure S28, Supporting Information). The discharge curves for various current densities ranging from 10 to 100 mW cm^−2^ shown in Figure 4e demonstrate that the Fe‐NP‐Cl‐C‐based battery delivers stable discharge potentials and exhibits a higher discharging voltage than Pt/C at 100 mW cm^−2^, indicating its exceptional rate performance. The discharge potential of the Fe‐NP‐Cl‐C‐based battery can resume to 1.35 V when the current density is returned to 10 mW cm^−2^, highlighting its outstanding reversibility attributed to the excellent ORR performance and stability of Fe‐NP‐Cl‐C. Furthermore, the Fe‐NP‐Cl‐C‐based battery exhibits a substantial specific capacity of 812 mAh g^−1^ at a current density of 10 mA cm^−2^, surpassing that of Pt/C which achieves a capacity of 705 mAh g^−1^ (Figure 4f). In a 175 h long‐term cycling test, the Fe‐NP‐Cl‐C‐based battery shows intensified charge and discharge potentials compared with Pt/C and Fe‐NP‐C catalysts (Figure S29, Supporting Information). Accordingly, the Fe‐NP‐Cl‐C‐based battery outperforms most of the recently reported Zn‐air batteries, underscoring its superior electrocatalytic performance and practicality for rechargeable Zn‐air batteries (Table S8, Supporting Information). The high intrinsic activity, hierarchical porous nanosheet structure, and hydrophobic character of Fe‐NP‐Cl‐C should account for the outstanding Zn‐air battery performance. On the one hand, the superior ORR activity and good OER activity of Fe‐N_2_P_2_‐Cl moiety provide the basis for superior oxygen electrocatalytic efficiency. On the other hand, the hierarchical porous nanosheet structure and hydrophobic character can greatly enhance the three‐phase boundaries at the air electrode and provide a highway for oxygen diffusion and electron transfer, guaranteeing fantastic device performance.

### 2.3 In situ Characterization and Density Functional Theory (DFT) Analysis

**Figure 5:**
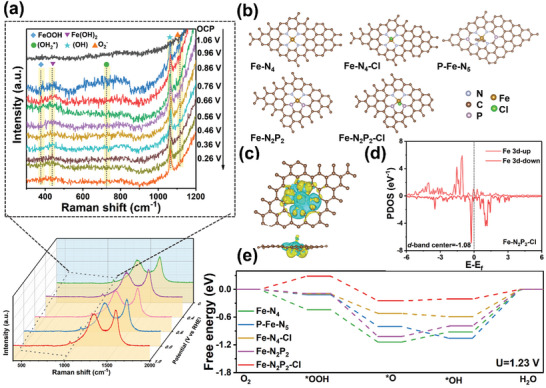
a) In situ Raman spectra of Fe‐NP‐Cl‐C in O_2_‐saturated 0.1 M KOH. b) Structural models of Fe‐N_4_, Fe‐N_4_‐Cl, P‐Fe‐N_5_, Fe‐N_2_P_2,_ and Fe‐N_2_P_2_‐Cl from the top view. c) Charge density difference of Fe‐N_2_P_2_‐Cl. Electron accumulation is in blue and depletion is in yellow. d) PDOS of Fe‐N_2_P_2_‐Cl. e) Free energy diagrams of ORR over Fe‐N_4_, Fe‐N_4_‐Cl, P‐Fe‐N_5_, Fe‐N_2_P_2_, and Fe‐N_2_P_2_‐Cl models in alkaline media at U = 1.23 V.

The reaction mechanism and catalytic intermediates over Fe‐NP‐Cl‐C were further investigated using in situ Raman spectra. **Figure** **5**a shows the Raman spectra of Fe‐NP‐Cl‐C at varying potentials in a 0.1 M KOH electrolyte. The *I*_D_/*I*_G_ intensity remains consistent across different potentials, signifying the excellent structural stability of Fe‐NP‐Cl‐C. No additional Raman peaks can be observed within the 300 to 1200 cm^−1^ range at the initial state, as the ORR process has not commenced yet. When the potential is changed to 1.06 V, a distinct peak emerges at ≈396 cm^−1^, which can be ascribed to FeOOH.^[^ ^29^ ^]^ This result elucidates a metamorphosis from O_2_ to OOH* at the atomic Fe‐N_2_P_2_‐Cl active sites. Further switching of the potential to 0.56 V yields a fresh peak at ≈441 cm^−1^ classified as Fe(OH)_2_, corroborating the conversion of OOH* to OH* on Fe‐NP‐Cl‐C.^[^ ^30^ ^]^ Additionally, peaks at ≈730 and 1060 cm^−1^ can be observed, corresponding to the O‐O stretching of OOH and the OH deformation mode, respectively.^[^ ^31^ ^]^ The obvious Raman peak near 1150 cm^−1^ is attributed to O‐O stretching vibrations of superoxide O^2−^ at 0.86 V, evidencing an interaction between Fe active sites and superoxide ion O^2−^.^[^ ^32^ ^]^

Density functional theory (DFT) calculations were performed to further shed light on the distinguished ORR performance of Fe‐NP‐Cl‐C. Based on the results of XPS and XAS, models with Fe‐N_2_P_2_‐Cl and P‐Fe‐N_5_ moieties were constructed for Fe‐NP‐Cl‐C and Fe‐NP‐C, respectively. To further elucidate the influence of P and Cl on the coordination environment and catalytic mechanism, a series of structural models including Fe‐N_4_, Fe‐N_4_‐Cl, and Fe‐N_2_P_2_ were constructed as controls (Figure 5b). Charge density difference diagrams of the optimal Fe‐N_2_P_2_‐Cl (Figure 5c) and other models (Figures S30–S32, Supporting Information) show significant redistribution of electron density around the Fe active centers. Further Bader charge analysis reveals noticeable charge transfers for the Fe‐N_4_, Fe‐N_4_‐Cl, Fe‐N_2_P_2_, and Fe‐N_2_P_2_‐Cl configurations (Figure S33, Supporting Information). Compared to traditional Fe‐N_4_ coordination (−0.92 e), the introduction of an electron with a drawing Cl atom (Fe‐N_4_‐Cl) makes the charge of the Fe center more positive (−1.11 e). When two N atoms of Fe‐N_4_ are replaced with P atoms (Fe‐N_2_P_2_), the P atoms show obvious charge donating to Fe (−0.57 e) and peripheral C atoms. Accordingly, the simultaneous coordination with P and Cl atoms can cooperatively modulate the electronic state of the Fe center (−0.80 e) for Fe‐N_2_P_2_‐Cl. The partial density of state (PODS) of the Fe displays an obvious upshift to −1.08 eV for Fe‐N_2_P_2_‐Cl configuration (Figure 5d) compared with traditional Fe‐N_4_ (Figure S34, Supporting Information), indicating the affluent unoccupied orbitals and stronger adsorption interaction with reaction intermediates.^[^ ^33^ ^]^ Based on the aforementioned in situ Raman results, the adsorption configurations of ORR intermediates (*OOH, *O, and *OH) adsorbed on Fe‐N_4_, Fe‐N_4_‐Cl, P‐Fe‐N_5_, Fe‐N_2_P_2_, and Fe‐N_2_P_2_‐Cl are optimized and illustrated in Figures S35–S39; Figure S40 (Supporting Information) shows the free‐energy pathways and exhibits decreasing trends at U = 0 V, indicating the thermodynamic favorability of the four‐electron ORR process. At U = 1.23 V, with the introduction of P, the Fe‐N_2_P_2_ configuration reveals an optimal *OOH adsorption (−0.09 eV) compared with traditional Fe‐N_4_ (Figure 5e). Whereas the desperation step of *OH demonstrates a smaller energy barrier for Fe‐N_4_‐Cl (−0.59 eV) than that of Fe‐N_4_ (−0.92 eV), indicating that coordination with Cl can optimize the adsorption of *OH. Especially, the largest uphill for Fe‐N_4_ (−0.92 eV), Fe‐N_4_‐Cl (−0.59 eV), Fe‐N_2_P_2_ (−0.79 eV), and P‐Fe‐N_5_ (−1.05 eV) configurations is the OH reduction step, demonstrating the potential determination process. Owing to the synergistic effect of P and Cl and the appropriate electron density of the Fe active center, the Fe‐N_2_P_2_‐Cl coordination requires a dramatically reduced energy barrier of 0.21 eV, suggesting its superior ORR activity to facilitate the conversion to H_2_O. It is worth mentioning that the limiting process shifts to the *OOH formation step, with a rather low energy barrier of 0.28 eV. Therefore, compared with congeneric Fe‐N_x_ single‐atom catalysts, both P and Cl atoms play significant roles in manipulating the local electron distribution of active centers and optimizing the adsorption of reaction intermediates, resulting in significantly reduced energy barriers and promoting reaction kinetics for ORR.

## 3 Conclusions

In summary, we have successfully demonstrated a feasible salt effect‐induced strategy to fabricate an advanced Fe‐NP‐Cl‐C single‐atom catalyst for ORR and Zn‐air batteries. The salt effect efficiently facilitates the formation of Fe‐P and Fe‐Cl bonds, leading to N, and P‐coordinated atomically dispersed Fe centers with extra‐axial Cl coordination. The resulting Fe‐N_2_P_2_‐Cl configuration with optimized charge distribution affords significantly reduced energy barriers and promoted ORR kinetics, demonstrating superior intrinsic oxygen electrocatalytic activity. Meanwhile, the salt effect induces the construction of an interlinked carbon nanosheet matrix with hierarchical pores, which facilitates the exposure of active sites and provides rapid oxygen diffusion and electron transfer channels at the three‐phase reaction interfaces. Consequently, Fe‐NP‐Cl‐C delivers a remarkable ORR activity with an E_1/2_ of 0.92 V that is superior to commercial Pt/C. As the air electrode, the Zn‐air battery based on Fe‐NP‐Cl‐C reveals an extremely high peak power density of 260 mW cm^−2^ and a large specific capacity of 812 mA h g^−1^, standing out among the reported congeneric catalysts. This study emphasizes the significance of salt effect on structural modification and coordination environment for single‐atom catalysts, providing a new perspective for engineering efficient oxygen electrocatalysts and energy conversion devices.

## Conflict of Interest

The authors declare no conflict of interest.

## Supporting information

Supporting Information

## Data Availability

The data that support the findings of this study are available in the supplementary material of this article.

## References

[1] a) X. Zhao , D. He , B. Y. Xia , Y. Sun , B. You , Adv. Mater. 2023, 35, 2210703; 10.1002/adma.202210703 36799551

[2] a) F. Wang , R. Zhang , Y. Zhang , Y. Li , J. Zhang , W. Yuan , H. Liu , F. Wang , H. L. Xin , Adv. Funct. Mater. 2023, 33, 2213863;

[3] a) Q. Sun , X.‐H. Li , K.‐X. Wang , T.‐N. Ye , J.‐S. Chen , Energy Environ. Sci. 2023, 16, 1838;

[4] a) Z. Wang , M. Cheng , Y. Liu , Z. Wu , H. Gu , Y. Huang , L. Zhang , X. Liu , Angew. Chem. Int. Ed. Engl. 2023, 62, 202301483; 10.1002/anie.202301483 36890120

[5] a) M. Fan , Q. Yuan , Y. Zhao , Z. Wang , A. Wang , Y. Liu , K. Sun , J. Wu , L. Wang , J. Jiang , Adv. Mater. 2022, 34, 2107040; 10.1002/adma.202107040 35038356

[6] a) Y. Li , Y. Ding , B. Zhang , Y. Huang , H. Qi , P. Das , L. Zhang , X. Wang , Z.‐S. Wu , X. Bao , Energy Environ. Sci. 2023, 16, 2629;

[7] a) Y. Chen , R. Gao , S. Ji , H. Li , K. Tang , P. Jiang , H. Hu , Z. Zhang , H. Hao , Q. Qu , X. Liang , W. Chen , J. Dong , D. Wang , Y. Li , Angew. Chem. Int. Ed. Engl. 2021, 60, 3212; 33124719 10.1002/anie.202012798

[8] L. Li , S. Huang , R. Cao , K. Yuan , C. Lu , B. Huang , X. Tang , T. Hu , X. Zhuang , Y. Chen , Small 2022, 18, 2105387. 10.1002/smll.202105387 34799983

[9] Y. Zhou , R. Lu , X. Tao , Z. Qiu , G. Chen , J. Yang , Y. Zhao , X. Feng , K. Müllen , J. Am. Chem. Soc. 2023, 145, 3647. 36744313 10.1021/jacs.2c12933 PMC9936543

[10] A. E. Thorarinsdottir , D. P. Erdosy , C. Costentin , J. A. Mason , D. G. Nocera , Nat. Catal. 2023, 6, 425.

[11] H. Jin , Z. Xu , Z.‐Y. Hu , Z. Yin , Z. Wang , Z. Deng , P. Wei , S. Feng , S. Dong , J. Liu , S. Luo , Z. Qiu , L. Zhou , L. Mai , B.‐L. Su , D. Zhao , Y. Liu , Nat. Commun. 2023, 14, 1518. 36934107 10.1038/s41467-023-37268-4 PMC10024750

[12] X. Tang , Y. Wei , W. Zhai , Y. Wu , T. Hu , K. Yuan , Y. Chen , Adv. Mater. 2023, 35, 2208942. 10.1002/adma.202208942 36349885

[13] a) T. T. Gu , D. T. Zhang , Y. Yang , C. Peng , D. F. Xue , C. Y. Zhi , M. Zhu , J. Liu , Adv. Funct. Mater. 2022, 33, 2212299;

[14] a) C. Jiao , Z. Xu , J. Shao , Y. Xia , J. Tseng , G. Ren , N. Zhang , P. Liu , C. Liu , G. Li , S. Chen , S. Chen , H.‐L. Wang , Adv. Funct. Mater. 2023, 33, 2213897;

[15] a) M. V. A. Queirós , W. Loh , J. Phys. Chem. B 2021, 125, 2968; 33720730 10.1021/acs.jpcb.0c11245

[16] J. Li , Y. Liu , X. Li , Q. Pan , D. Sun , L. Men , B. Sun , C. Xu , Z. Su , Chem. Eng. J. 2022, 431, 133695.

[17] a) X. Shao , M. Liang , M. G. Kim , S. Ajmal , A. Kumar , X. Liu , H. S. Jung , H. Jin , F. Cao , J. Yu , K. M. Tran , H. Ko , J. Lee , J. W. Bae , H. Lee , Adv. Funct. Mater. 2023, 33, 2211192;

[18] Y. Tian , Z. Wu , M. Li , Q. Sun , H. Chen , D. Yuan , D. Deng , B. Johannessen , Y. Wang , Y. Zhong , L. Xu , J. Lu , S. Zhang , Adv. Funct. Mater. 2022, 32, 2209273.

[19] L. Yan , B. Xie , C. Yang , Y. Wang , J. Ning , Y. Zhong , Y. Hu , Adv. Energy. Mater. 2023, 13, 2204245.

[20] a) X. Hai , S. Xi , S. Mitchell , K. Harrath , H. Xu , D. F. Akl , D. Kong , J. Li , Z. Li , T. Sun , H. Yang , Y. Cui , C. Su , X. Zhao , J. Li , J. Pérez‐Ramírez , J. Lu , Nat. Nanotechnol. 2022, 17, 174; 34824400 10.1038/s41565-021-01022-y

[21] Y. Xiong , H. Li , C. Liu , L. Zheng , C. Liu , J.‐O. Wang , S. Liu , Y. Han , L. Gu , J. Qian , D. Wang , Adv. Mater. 2022, 34, 2110653. 10.1002/adma.202110653 35263466

[22] a) K. Yuan , D. Lützenkirchen‐Hecht , L. Li , L. Shuai , Y. Li , R. Cao , M. Qiu , X. Zhuang , M. K. H. Leung , Y. Chen , U. Scherf , J. Am. Chem. Soc. 2020, 142, 2404; 31902210 10.1021/jacs.9b11852

[23] a) S. Ding , J. A. Barr , Q. Shi , Y. Zeng , P. Tieu , Z. Lyu , L. Fang , T. Li , X. Pan , S. P. Beckman , D. Du , H. Lin , J.‐C. Li , G. Wu , Y. Lin , ACS Nano 2022, 16, 15165; 36094168 10.1021/acsnano.2c06459

[24] J.‐X. Peng , W. Yang , Z. Jia , L. Jiao , H.‐L. Jiang , Nano Res. 2022, 15, 10063.

[25] a) C. Xin , W. Shang , J. Hu , C. Zhu , J. Guo , J. Zhang , H. Dong , W. Liu , Y. Shi , Adv. Funct. Mater. 2022, 32, 2108345.

[26] a) L. Zong , K. Fan , P. Li , F. Lu , B. Li , L. Wang , Adv. Energy. Mater. 2023, 13, 2203611;

[27] a) B. Ji , J. Gou , Y. Zheng , X. Zhou , P. Kidkhunthod , Y. Wang , Q. Tang , Y. Tang , Adv. Mater. 2022, 34, 2202714; 10.1002/adma.202202714 35522047

[28] a) J. Liu , Y. Liu , P. Li , L. Wang , H. Zhang , H. Liu , J. Liu , Y. Wang , W. Tian , X. Wang , Z. Li , M. Wu , Carbon 2018, 126, 1;

[29] M. Jiang , F. Wang , F. Yang , H. He , J. Yang , W. Zhang , J. Luo , J. Zhang , C. Fu , Nano Energy 2022, 93, 106793.

[30] J. Wei , D. Xia , Y. Wei , X. Zhu , J. Li , L. Gan , ACS Catal. 2022, 12, 7811.

[31] M. Li , H. Zhu , Q. Yuan , T. Li , M. Wang , P. Zhang , Y. Zhao , D. Qin , W. Guo , B. Liu , X. Yang , Y. Liu , Y. Pan , Adv. Funct. Mater. 2022, 33, 2210867.

[32] Y. Wang , K. Li , R. Cheng , Q. Xue , F. Wang , Z. Yang , P. Meng , M. Jiang , J. Zhang , C. Fu , Chem. Eng. J. 2022, 450, 138213.

[33] a) L. Hu , C. Dai , L. Chen , Y. Zhu , Y. Hao , Q. Zhang , L. Gu , X. Feng , S. Yuan , L. Wang , B. Wang , Angew. Chem. Int. Ed. Engl. 2021, 60, 27324; 34704324 10.1002/anie.202113895

