# Supplementary material for: Salt Effect Engineering Single Fe‐N2P2‐Cl Sites on Interlinked Porous Carbon Nanosheets for Superior Oxygen Reduction Reaction and Zn‐Air Batteries
Source: Adv Sci (Weinh). 2024 Jan 15;11(12):2306599. doi: 10.1002/advs.202306599 (PMC10966546; doi:10.1002/advs.202306599)
Supplement: Supplementary file 1 — Supporting Information [file ADVS-11-2306599-s001.pdf]

ADVANCED SCIENCE
Open Access

Supporting Information

for *Adv. Sci.*, DOI 10.1002/advs.202306599

Salt Effect Engineering Single Fe-N_2P_2-Cl Sites on Interlinked Porous Carbon Nanosheets for Superior Oxygen Reduction Reaction and Zn-Air Batteries

Xiaojie Tan, Jinqiang Zhang, Fengliang Cao, Yachao Liu, Hao Yang, Qiang Zhou, Xudong Li, Rui Wang, Zhongtao Li, Han Hu, Qingshan Zhao and Mingbo Wu**

Supporting Information

Salt Effect Engineering Single Fe-N_2P_2-Cl Sites on Interlinked Porous Carbon Nanosheets for Superior Oxygen Reduction Reaction and Zn-Air Batteries

Xiaojie Tan, Jinqiang Zhang, Fengliang Cao, Yachao Liu, Hao Yang, Qiang Zhou, Xudong Li, Rui Wang, Zhongtao Li, Han Hu, Qingshan Zhao, and Mingbo Wu**

1. Experimental Details

1.1. Materials

Sodium chloride (NaCl, 99.5 wt.%), phytic acid (PA, 70 wt.% solution), iron nitrate nonahydrate ($Fe(NO)_3 \cdot 9H_2O$, 99.9 wt.%), o-phenylenediamine (OPD, 98 wt.%), hydrochloric acid (HCl, 36-38 wt.%), potassium hydroxide (KOH 95 wt.%), potassium thiocyanate (KSCN 99 wt. %), Nafion solution (5 wt.%) and 20 wt.% Pt/C catalyst were purchased from Aladdin Biochemical Technology Co., Ltd. The chemical reagents were used directly without further purification.

1.2. Synthesis of the Electrocatalysts

943 mg PA was dispersed in 16 mL water, and 4 g NaCl was added into the solution to promote the ionization of PA. 2.475 g $Fe(NO)_3 \cdot 9H_2O$ was added with stirring for 3 h to chelate with PA to obtain SE-PA-Fe. Then 325 mg OPD and 5mL H_2O was added into the solution and assembled with SE-PA-Fe to achieve SE-PA-Fe-OPD intermediate. The resulted suspension was filtered and washed deionized water 3 times to remove the NaCl salt completely. The mixture was dried at 60 °C and subsequently carbonized at 900 °C for 2 h (with a heating rate of 5 °C min^{-1}) under N_2 atmosphere. The obtained sample was treated with 1 M HCl at 90 °C for 24 h, and the precipitate was rinsed with deionized water to neutral and dried to obtain Fe-NP-Cl-C. As a control, Fe-NP-C and Fe-N-Cl-C was prepared with the same method except for the addition of NaCl salt and PA, respectively. Whereas Fe-N-C was fabricated without the inclusion of NaCl and PA. To investigate the influence of NaCl dosage, Fe-NP-Cl-C-3 and Fe-NP-Cl-C-5 were synthesized by changing to 3 g NaCl and 5 g NaCl, respectively. To distinguish the effect

of salt, various Fe-NP-Cl-C-LiCl, Fe-NP-F-C-NaF, Fe-NP-C-$NaNO_3$, Fe-NP-Br-C-NaBr, Fe-NP-Cl-C-KCl and Fe-NP-Cl-C-$MgCl_2$ samples were prepared by changing NaCl into equimolar LiCl, NaF, $NaNO_3$, NaBr, KCl and $MgCl_2$, respectively.

1.3. Material Characterization

Scanning electron microscopy (SEM) images (Hitachi S-4800) and transmission electron microscopy (TEM) images (JEM-2100F) were captured to observe the microstructures and morphologies of samples. Energy dispersive X-ray (EDX) mapping were recorded on the JEM-2100 microscope operating at 200 kV. High-angle annular dark-field scanning transmission electron microscopy (HAADF-STEM) images were carried out on a spherical aberration corrected Titan 80-300 operated at 300 kV. Raman analysis was conducted on a Jobin-Yvon Labram-010 Raman spectrometer with a wavelength of 532 nm. X-ray photoelectron spectroscopy (XPS) was performed on a Kratos Axis Ultra equipment (Chestnut Ridge) with Mg K_α radiation (1486.6 eV). The specific surface area and pore structure were measured by the Brunauer-Emmett-Teller (BET) method on a sorptometer (Micromeritics, ASAP 2020). The metal loading was measured on by an inductively coupled plasma-atomic emission spectroscopy (ICP-AES, AGILENT730ES). X-ray absorption near edge structure (XANES) and extend X-ray absorption fine structure (EXAFS) were performed at 1W1B station in Beijing Synchrotron Radiation Facility (BSRF).

1.4. Electrochemical Measurements

Electrochemical measurements were performed on an electrochemical workstation (CHI 760E) with a three-electrode cell system, using an Ag/AgCl (saturated KCl) electrode as reference electrode and carbon rod as the counter electrode. All the potentials were referred to a reversible hydrogen electrode (RHE):

$$E_{RHE} = E_{Ag/AgCl} + 0.197\ V + 0.0591pH \qquad \text{Equation S1}$$

A rotating ring-disk electrode (RRDE) with a glassy carbon disk (GCE, 4 mm in diameter) and a Pt ring was loaded with the as-prepared catalysts, serving as the working electrode. 2 mg of samples were dispersed in 0.8 mL ethanol and 5 µL of 5 wt.% Nafion solution under sonication for 30 min. 15 µL of the suspension was pipetted onto the GCE and dried in air at a catalyst loading of 0.30 mg cm^{-2}. O_2 was saturated in the electrolyte before the tests. Cyclic voltammetry (CV) tests were conducted in an O_2 saturated 0.1 M KOH electrolyte at a scan rate of 0.1 V s^{-1}. Linear-sweep voltammetry (LSV) measurements were performed at a scan rate of 5 mV s^{-1} under an electrode rotation rate of 1600 rpm. The applied potential at the ring electrode is 0.5 V.

The peroxide percentage (H_2O_2%) and the electron transfer number (n) are determined by the following equations:

$$H_2O_2\%=200\times(I_R/N)/(I_D+I_R/N) \quad \text{(Equation S2)}$$

$$n=4\times I_D/(I_D+I_R/N) \quad \text{(Equation S3)}$$

where I_D is the disk current, I_R is the ring current, and N is current collection efficiency of the Pt ring. N is determined to be 0.40.

The ORR tests were performed in an O_2 saturated 0.1 M KOH aqueous solution (70 mL). As to the methanol tolerance test, 47.5 μL methanol dissolved in 1 mL of 0.1 M KOH was added to the O_2-saturated 0.1 M KOH aqueous solution at 300 s.

A primary Zn-air battery was assembled to verify the catalysts for application in energy conversion and storage devices. 2 mg catalyst was dispersed in 0.8 mL ethanol and 5 μL of 5 wt.% Nafion solution under sonication for 30 min to form a homogeneous catalyst ink. 410 μL of the suspension was loaded on the carbon cloth (1*1 cm^{-2}) and dried in air at a catalyst loading of 1 mg cm^{-2}. Polished zinc plate (with a thickness of 0.5 mm), 6 M KOH (containing 0.2 M $Zn(OAc)_2$) solution and carbon cloth loaded with 1 mg cm^{-2} catalyst were used as the negative electrode, electrolyte and positive electrode, respectively. Charge-discharge cycle experiments were carried out at 10 mA cm^{-2} for 30 minutes every cycle (15 mins charge, 15 mins discharge).

1.5. Density Functional Theory (DFT) calculations

Vienna Ab-initio Simulation Package was used to conduct all DFT calculations (VASP). The exchange correlation energy was computed using generalized gradient approximation (GGA) as parameterized by Perdew et al. based on the Perdew-Burke-Ernzerhof (PBE) functional.[1] In a plane wave basis with a kinetic cutoff energy of 500 eV, the Kohn-Sham orbitals were stretched. For graphite surfaces with unit cell of 5×5, Monkhorst-Pack meshes of 3×3×1 k-point samplings in the surface Brillouin zones were chosen based on the lattice size. The Monkhorst-Pack meshes of 3×3×1 k-point samplings in the surface Brillouin zones were used for the graphite surfaces with unit cell of 5×5. All of structures were completely relaxation. To eliminate periodic interactions, the 15 Å vacuum layer was used in the direction of the surface normal. For all computations, the optimization was converged until the stresses on each atom were less than 0.01 eV/ Å and the overall energy differences were less than 10^{-6} eV.

2. Supporting Figures and Tables

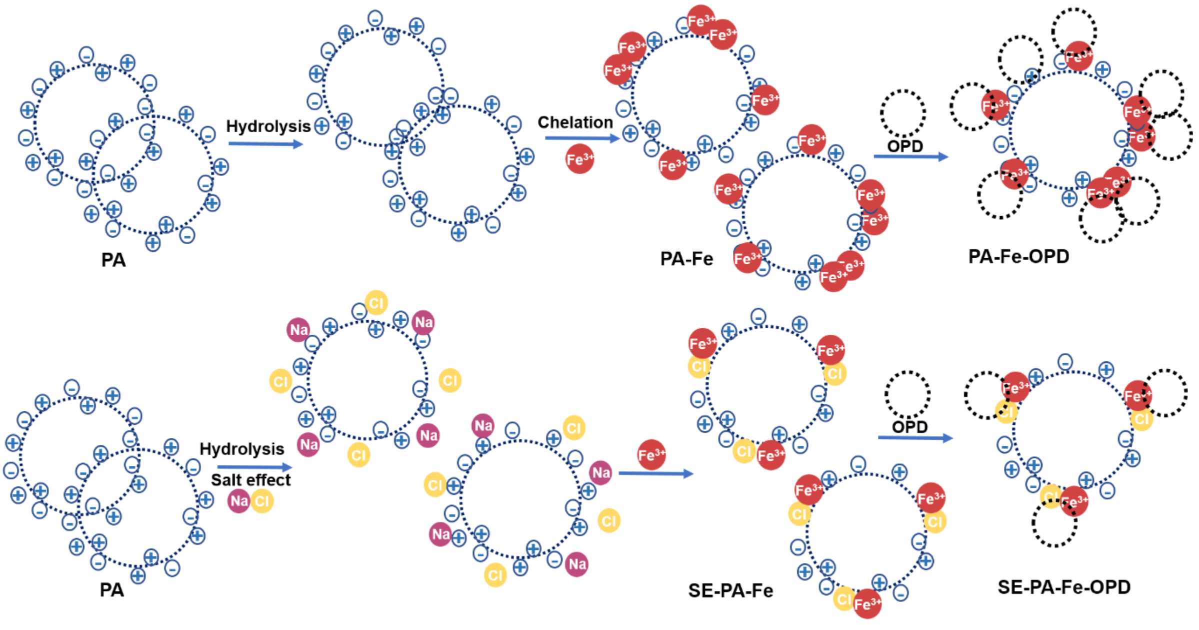

Scheme S1. Illustration for the formation mechanism of SE-PA-Fe and SE-PA-Fe-OPD induced by salt effect.

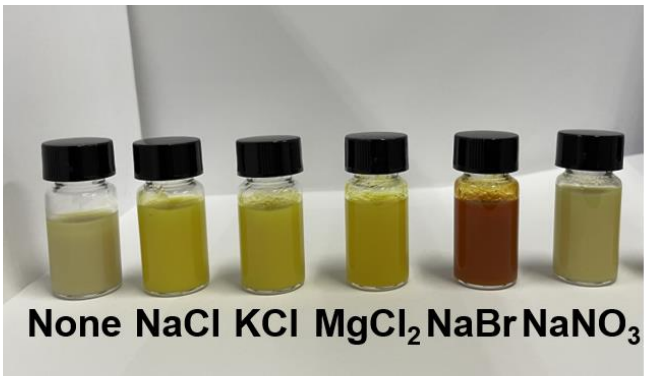

Figure S1. Photograph of 0-PA-Fe, SE-PA-Fe, SE-PA-Fe-NaCl, SE-PA-Fe-$MgCl_2$, SE-PA-Fe-NaBr and SE-PA-Fe-$NaNO_3$.

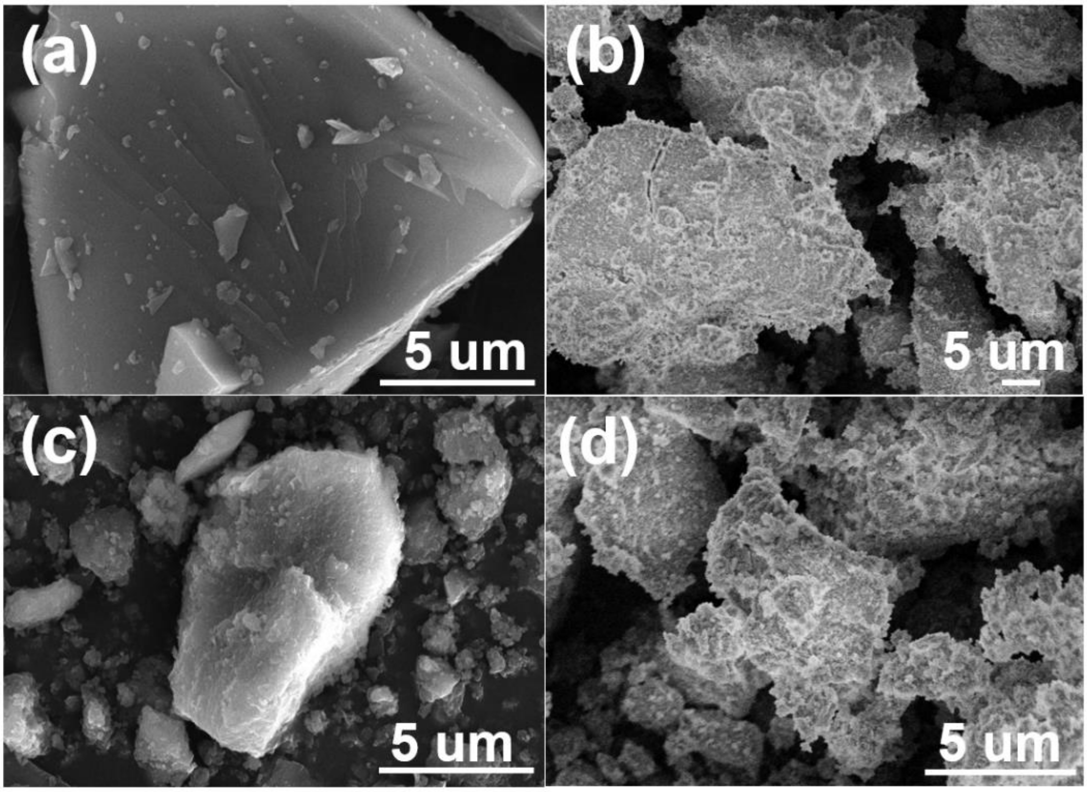

Figure S2. SEM images of (a) PA-Fe, (b) PA-Fe-OPD, (c) SE-PA-Fe, (d) SE-PA-Fe-OPD.

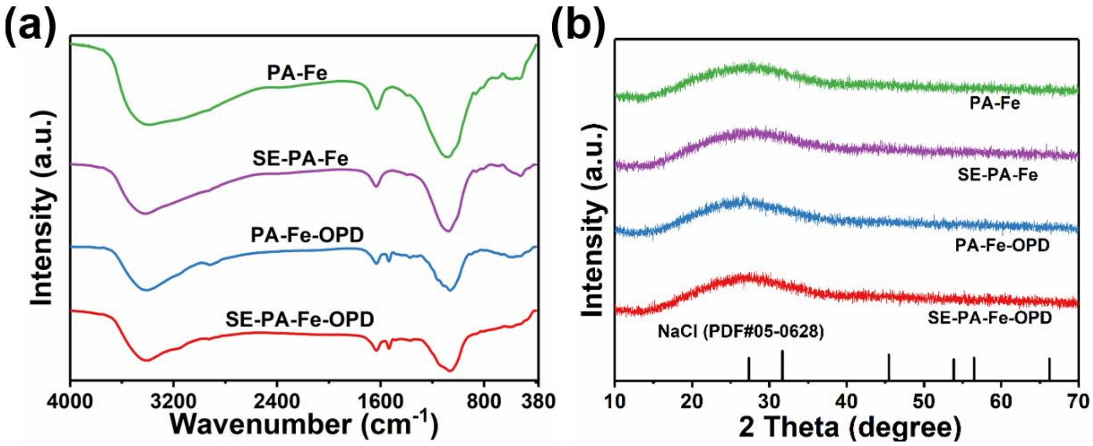

Figure S3. (a) FT-IR spectra and (b) XRD patterns of PA-Fe, SE-PA-Fe, PA-Fe-OPD and SE-PA-Fe-OPD.

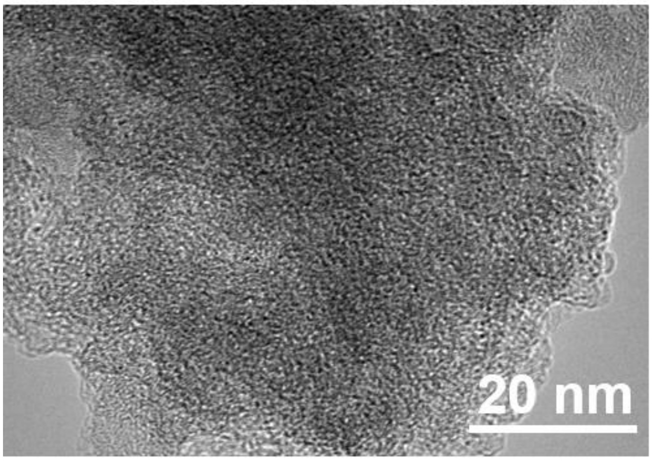

Figure S4. HR-TEM image of Fe-NP-C.

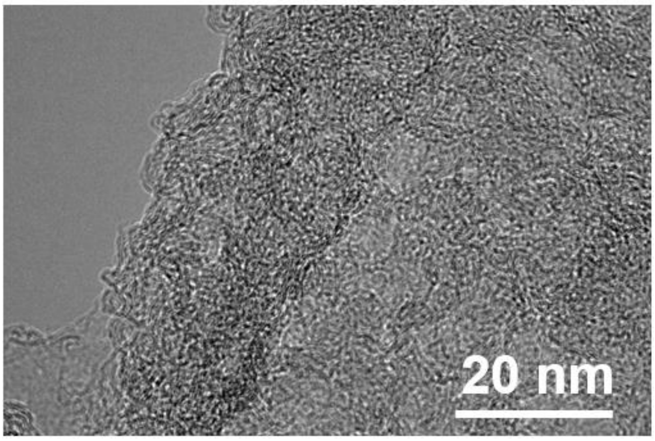

Figure S5. HR-TEM image of Fe-NP-Cl-C.

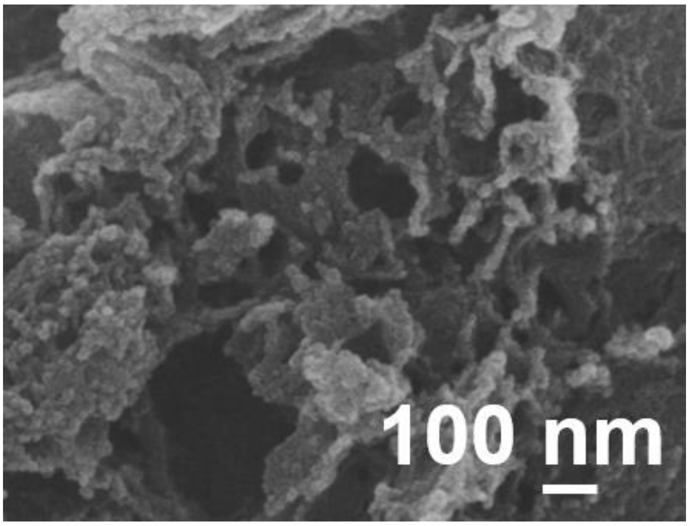

Figure S6. SEM image of Fe-NP-C-$NaNO_3$.

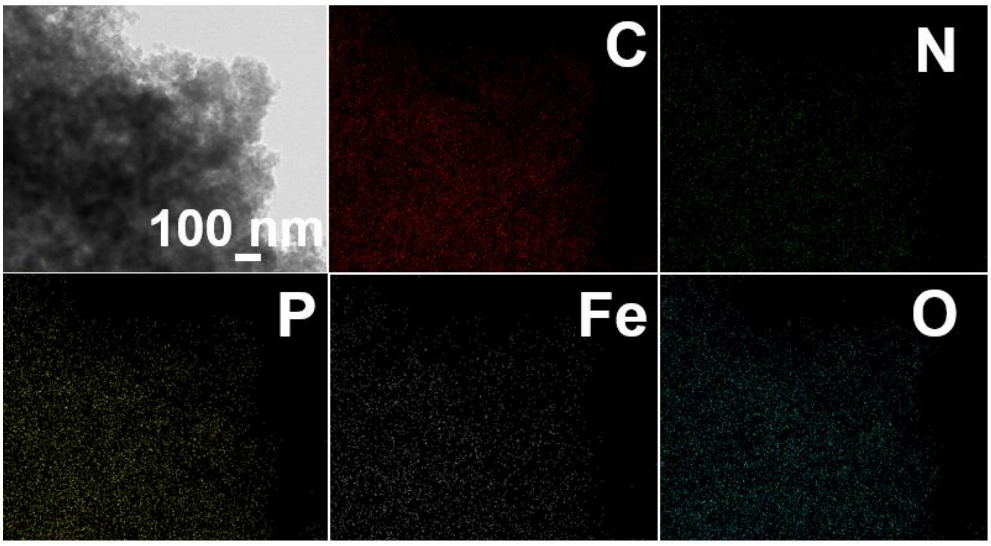

Figure S7. (a) TEM image of Fe-NP-C and (b) corresponding EDX elemental mapping of C, N, P and Fe.

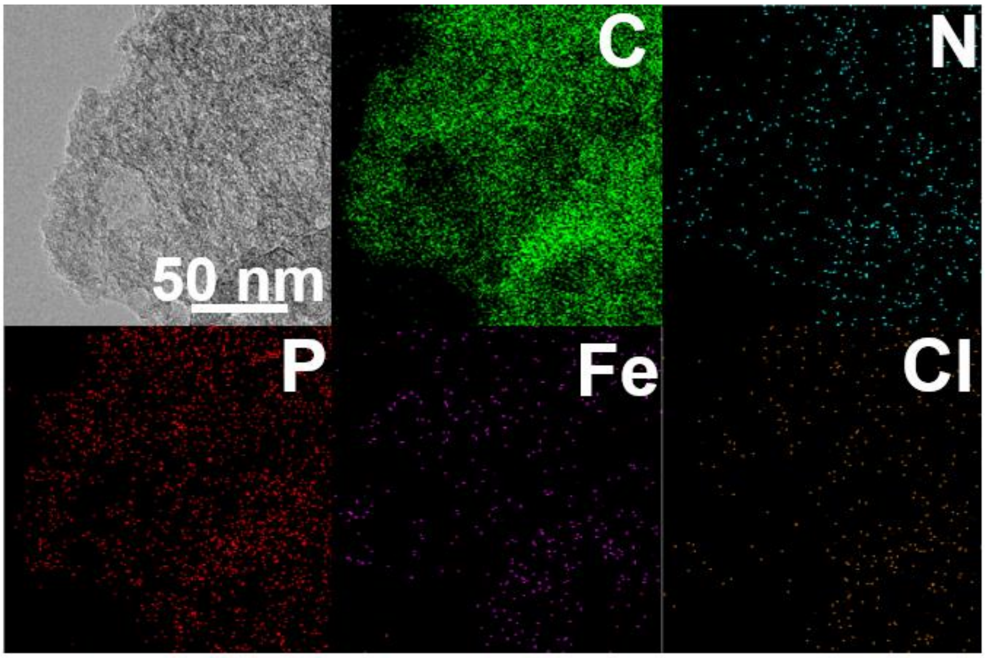

Figure S8. TEM image of Fe-NP-Cl-C and corresponding EDX elemental mapping of C, N, P, Fe and Cl.

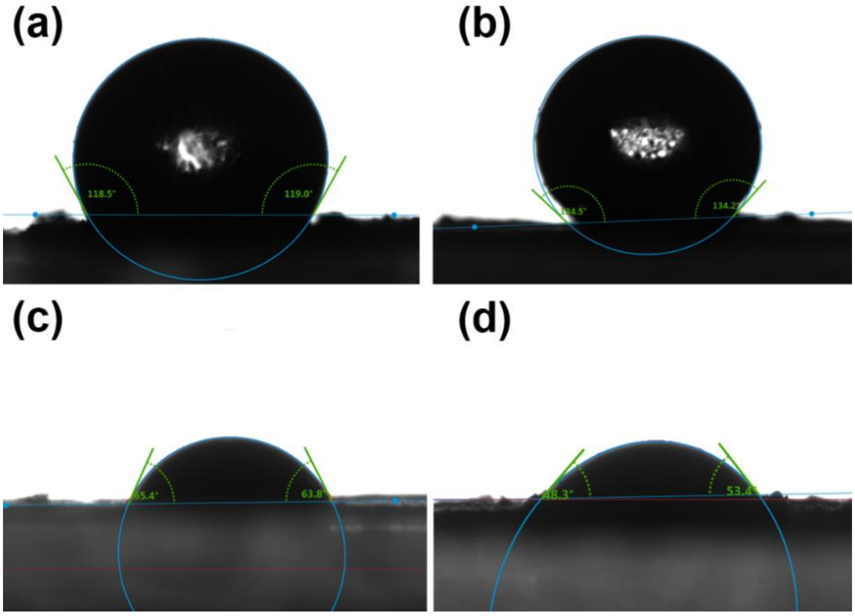

Figure S9. Water contact angles of (a) Fe-NP-C and (b) Fe-NP-Cl-C. O_2-saturated KOH solution contact angles of (c) Fe-NP-C and (d) Fe-NP-Cl-C.

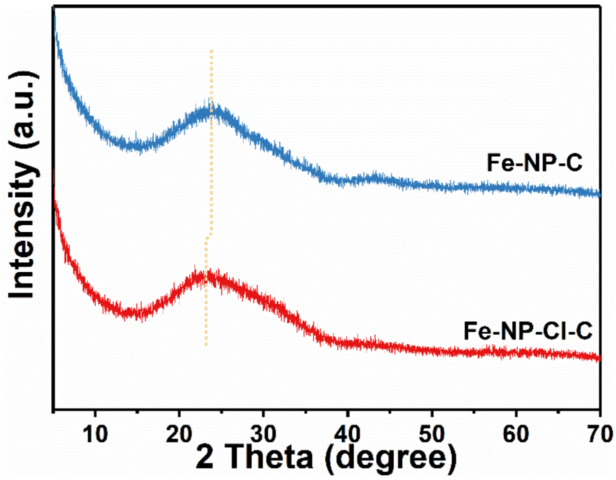

Figure S10. XRD patterns of Fe-NP-C and Fe-NP-Cl-C.

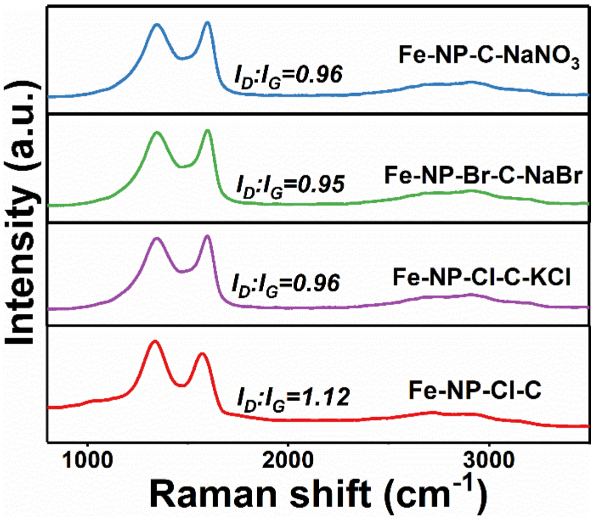

Figure S11. Raman spectra of Fe-NP-C-$NaNO_3$, Fe-NP-Br-C-NaBr, Fe-NP-Cl-C-KCl and Fe-NP-Cl-C.

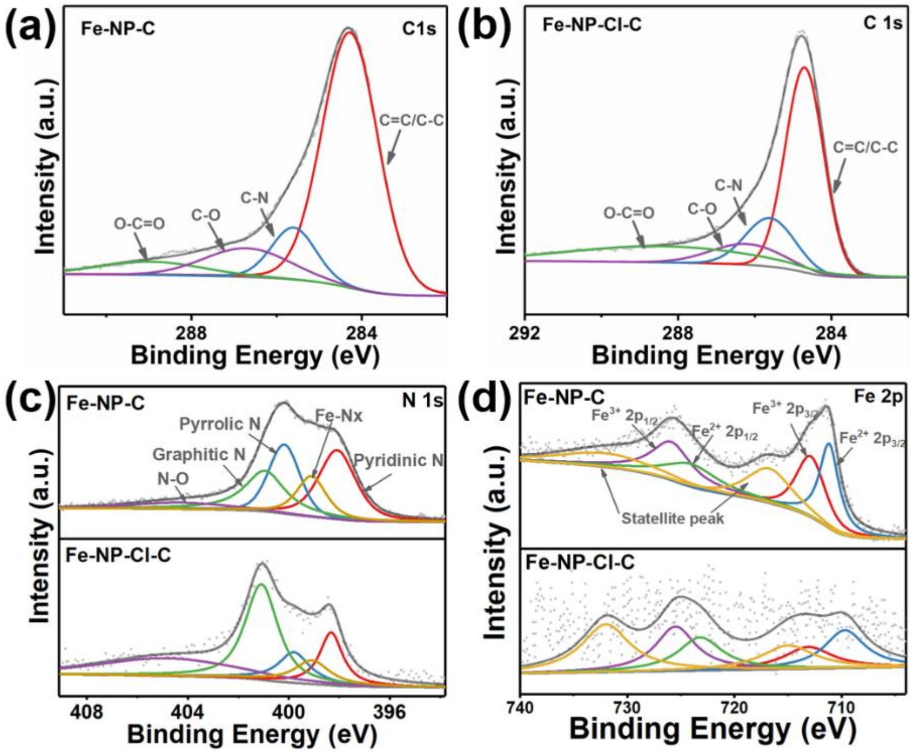

Figure S12. C 1s XPS spectra of (a) Fe-NP-C and (b) Fe-NP-Cl-C. (c) N 1s XPS spectra of (d) Fe 2p of Fe-NP-C and Fe-NP-Cl-C.

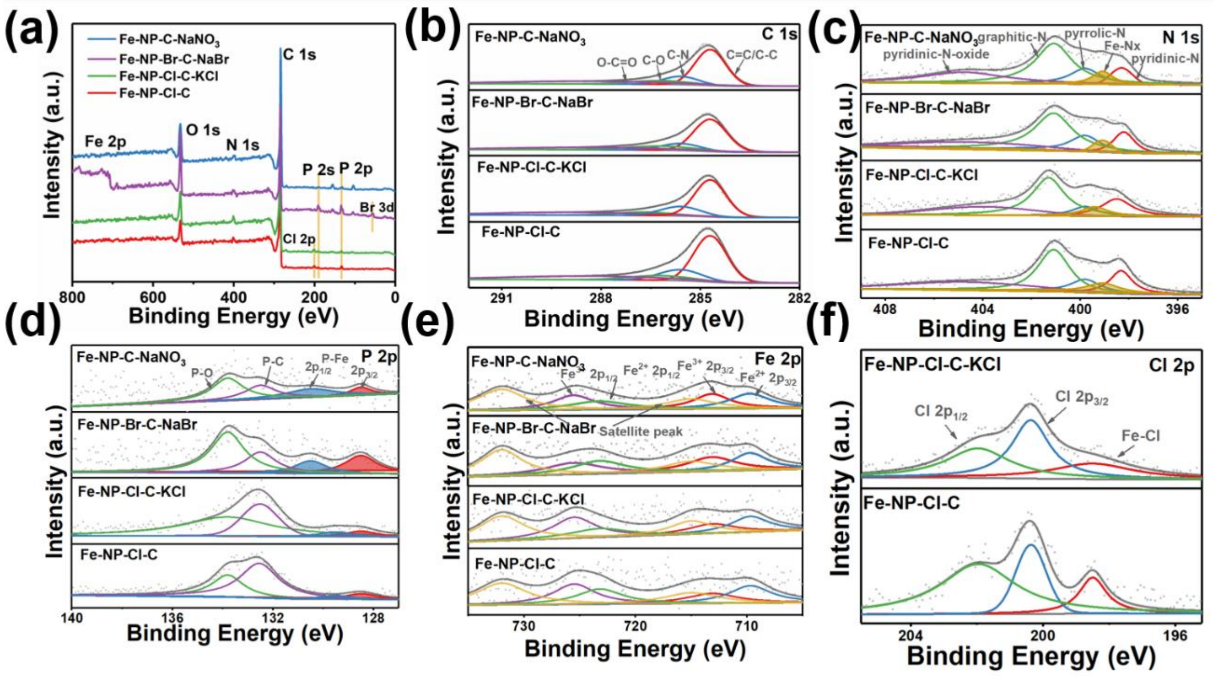

Figure S13. (a) XPS survey spectra of Fe-NP-C-$NaNO_3$, Fe-NP-Br-C-NaBr, Fe-NP-Cl-C-KCl and Fe-NP-Cl-C. (b) C 1s, (c) N 1s (d) P 2p, (e) Fe 2p XPS spectra of Fe-NP-C-$NaNO_3$, Fe-NP-Br-C-NaBr, Fe-NP-Cl-C-KCl and Fe-NP-Cl-C. (f) Cl 2p XPS spectra of Fe-NP-Cl-C-KCl and Fe-NP-Cl-C.

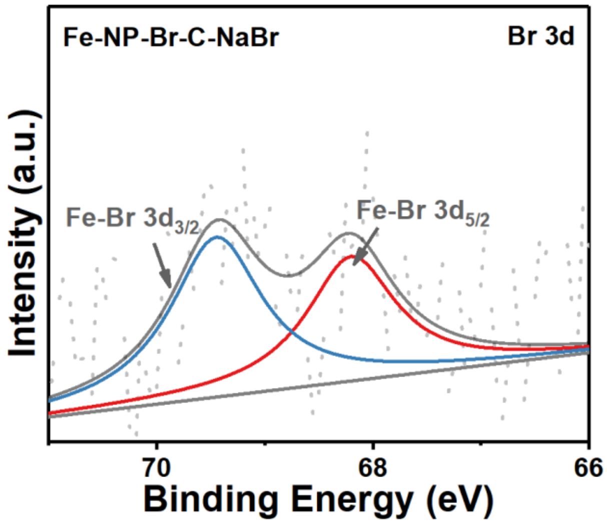

Figure S14. Br 3d XPS spectrum of Fe-NP-Br-C-NaBr.

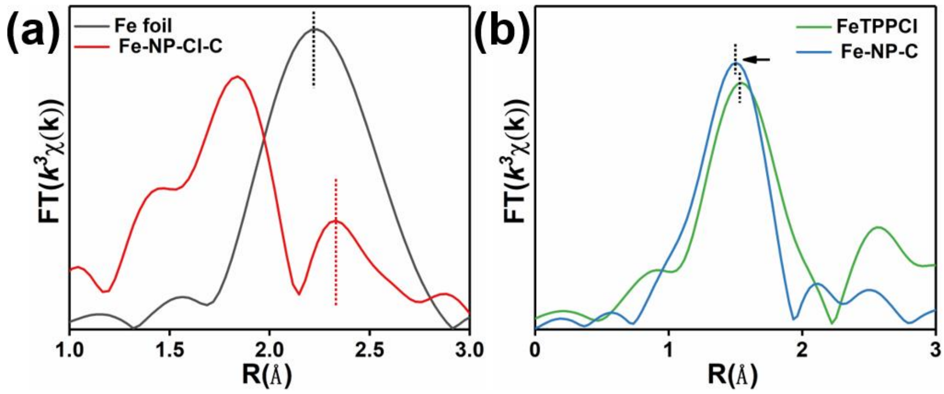

Figure S15. k^3-weighted FT spectra in R space for (a) Fe foil and (b) FeTPPCl and Fe-NP-C.

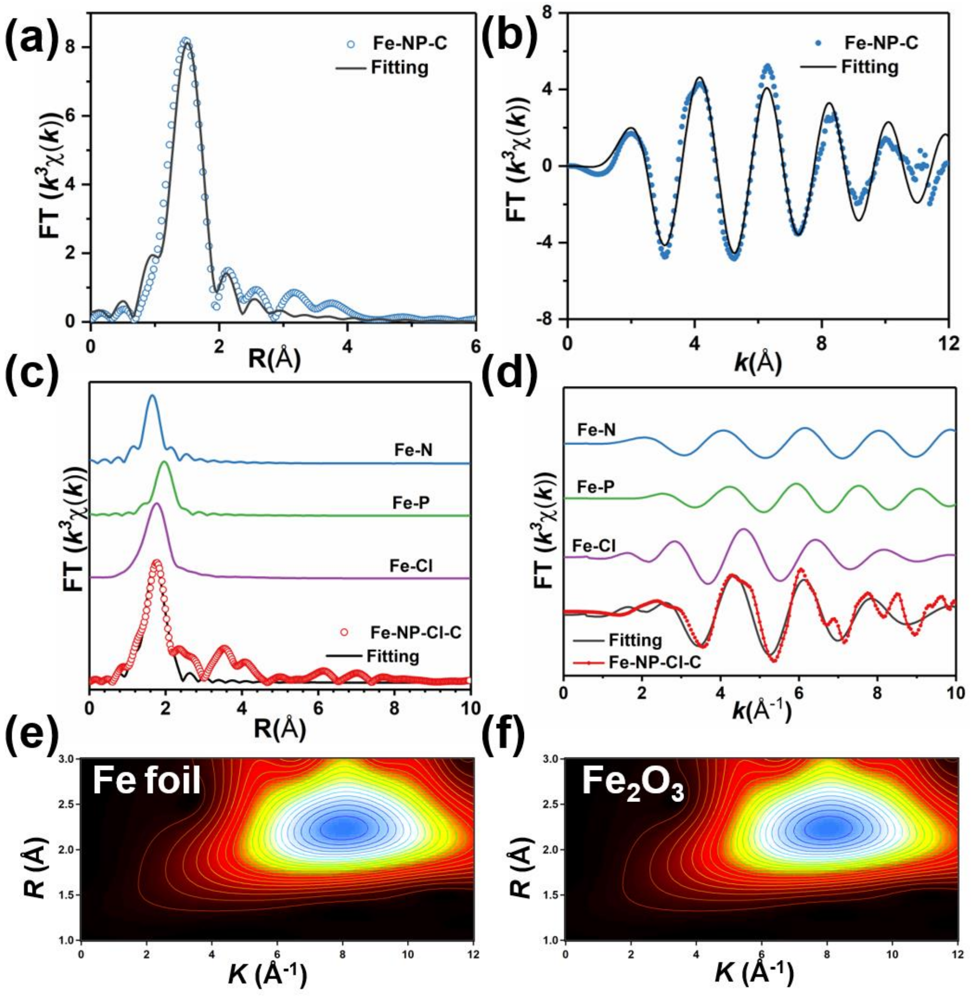

Figure S16. EXAFS fitting curves of (a, b) Fe-NP-C and (c, d) Fe-NP-Cl-C in the R space and k space. Wavelet transform plots of (e) Fe foil and (f) Fe_2O_3.

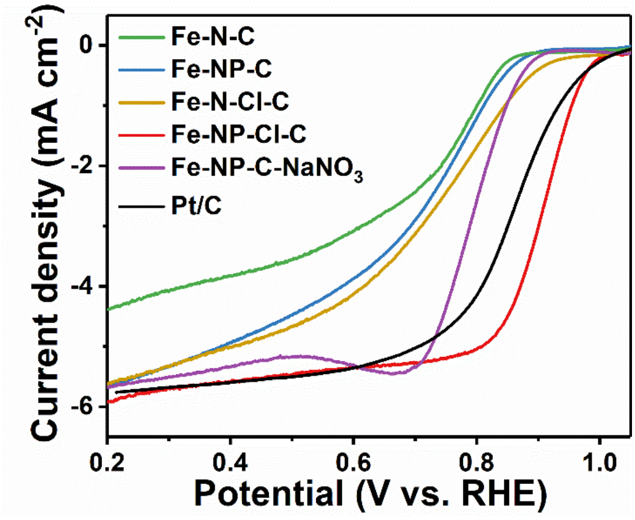

Figure S17. LSV curves of Fe-N-C, Fe-N-Cl-C, Fe-NP-C, Fe-NP-Cl-C and commercial Pt/C (20 wt.%) in O_2-saturated 0.1 M KOH solution at a rotating speed of 1600 rpm.

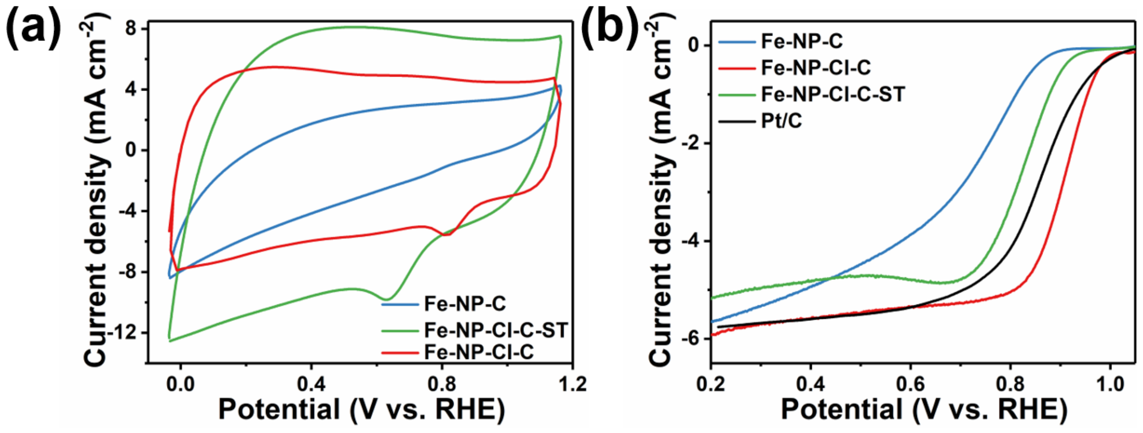

Figure S18. (a) CV curves of Fe-NP-C, Fe-NP-Cl-C-ST and Fe-NP-Cl-C in O_2-saturated 0.1 M KOH solution (b) LSV curves of Fe-NP-C, Fe-NP-Cl-C-ST, Fe-NP-Cl-C and commercial Pt/C (20 wt.%) in O_2-saturated 0.1 M KOH solution at a rotating speed of 1600 rpm.

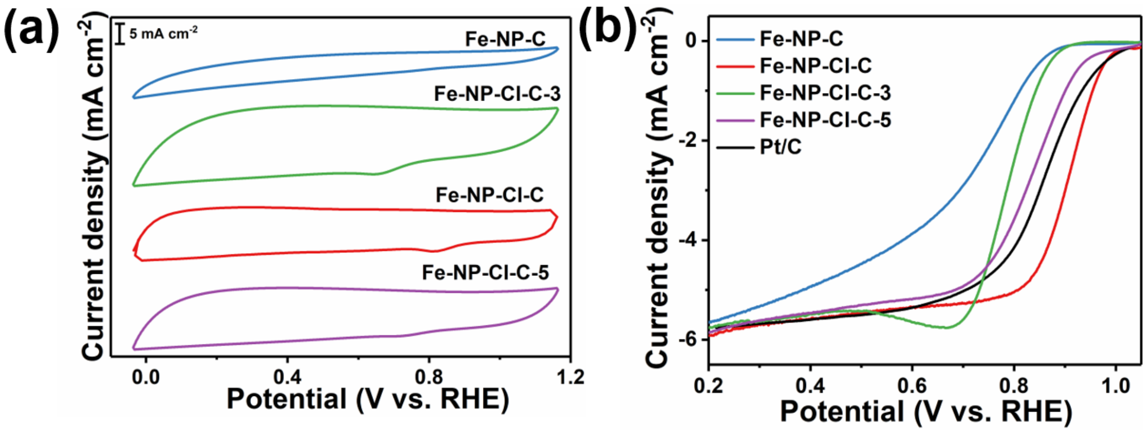

Figure S19. (a) CV curves of Fe-NP-C, Fe-NP-Cl-C-3, Fe-NP-Cl-C and Fe-NP-Cl-C-5 in O_2-saturated 0.1 M KOH solution (b) LSV curves of Fe-NP-C, Fe-NP-Cl-C-3, Fe-NP-Cl-C, Fe-NP-Cl-C-5 and commercial Pt/C (20 wt.%) in O_2-saturated 0.1 M KOH solution at a rotating speed of 1600 rpm.

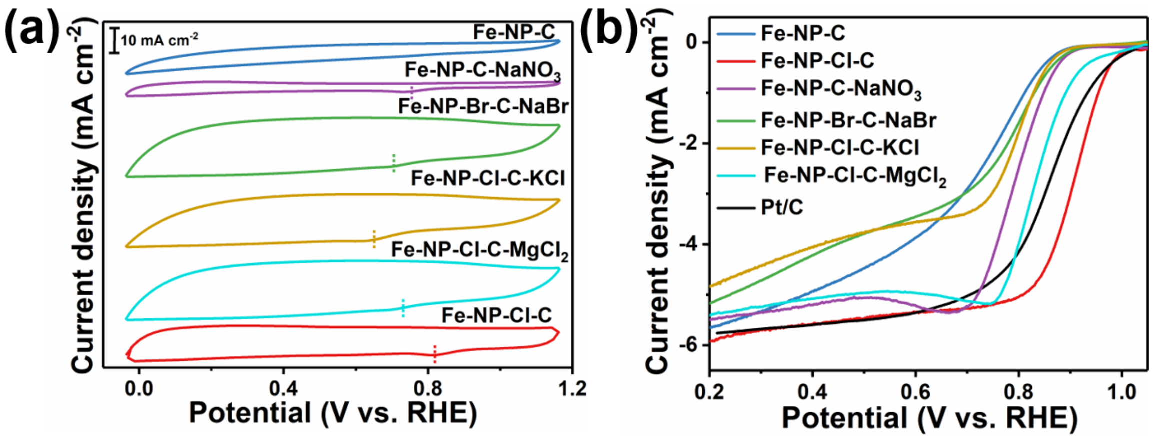

Figure S20. (a) CV curves of Fe-NP-C, Fe-NP-Cl-C, Fe-NP-C-$NaNO_3$, Fe-NP-Br-C-NaBr, Fe-NP-Cl-C-KCl and Fe-NP-Cl-C-$MgCl_2$ in O_2-saturated 0.1 M KOH solution (b) LSV curves of Fe-NP-C, Fe-NP-Cl-C, Fe-NP-C-$NaNO_3$, Fe-NP-Br-C-NaBr, Fe-NP-Cl-C-KCl, Fe-NP-Cl-C-$MgCl_2$ and commercial Pt/C (20 wt.%) in O_2-saturated 0.1 M KOH solution at a rotating speed of 1600 rpm.

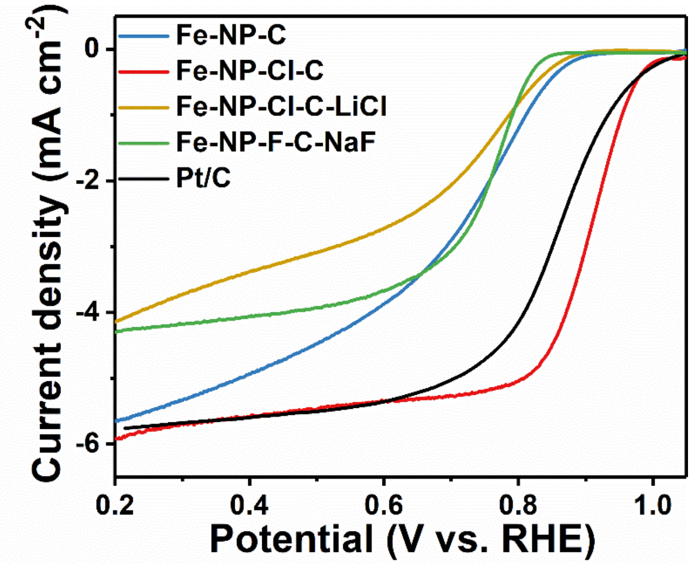

Figure S21. LSV curves of Fe-NP-C, Fe-NP-Cl-F-C-NaF, Fe-NP-Cl-C-LiCl, Fe-NP-Cl-C and commercial Pt/C (20 wt.%) in O_2-saturated 0.1 M KOH solution at a rotating speed of 1600 rpm.

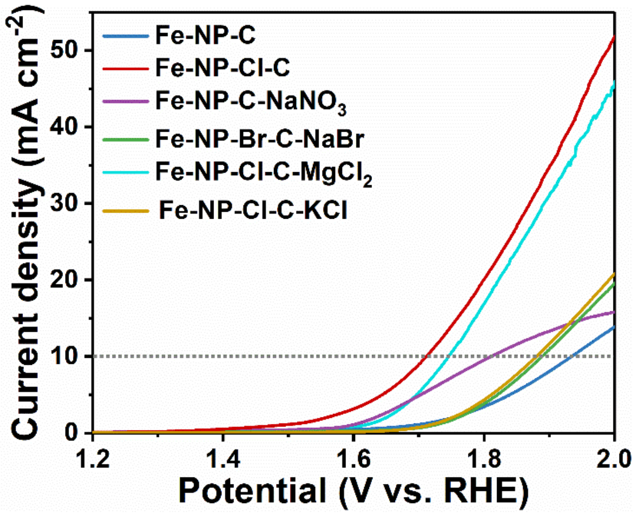

Figure S22. OER LSV curves of Fe-NP-C, Fe-NP-Cl-C, Fe-NP-C-$NaNO_3$, Fe-NP-Br-C-NaBr, Fe-NP-Cl-C-KCl, and Fe-NP-Cl-C-$MgCl_2$ in 1 M KOH.

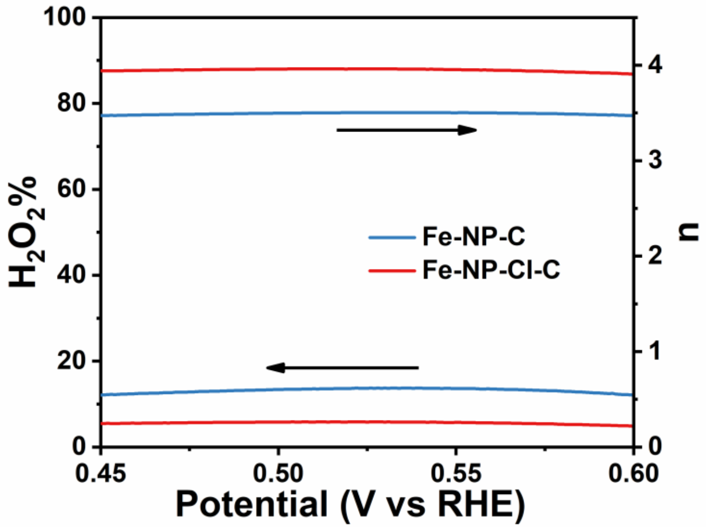

Figure S23. Electron transfer number and H_2O_2 yield at various potentials of Fe-NP-C and Fe-NP-Cl-C.

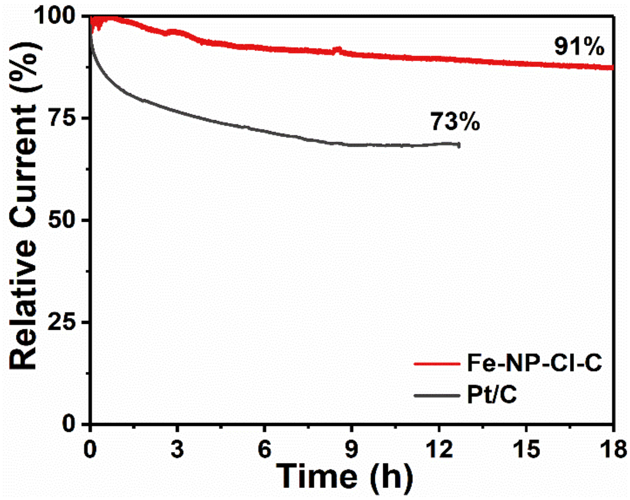

Figure S24. i-t curves for Fe-NP-Cl-C and Pt/C in O_2-saturated 0.1 M KOH solution.

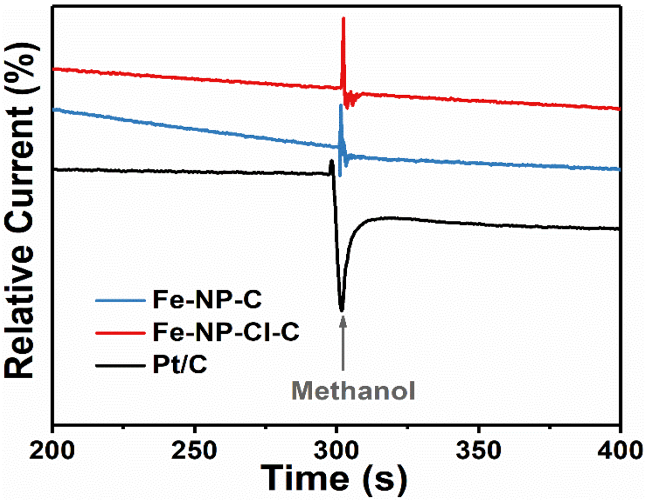

Figure S25. Methanol tolerance tests of Fe-NP-C, Fe-NP-Cl-C and Pt/C.

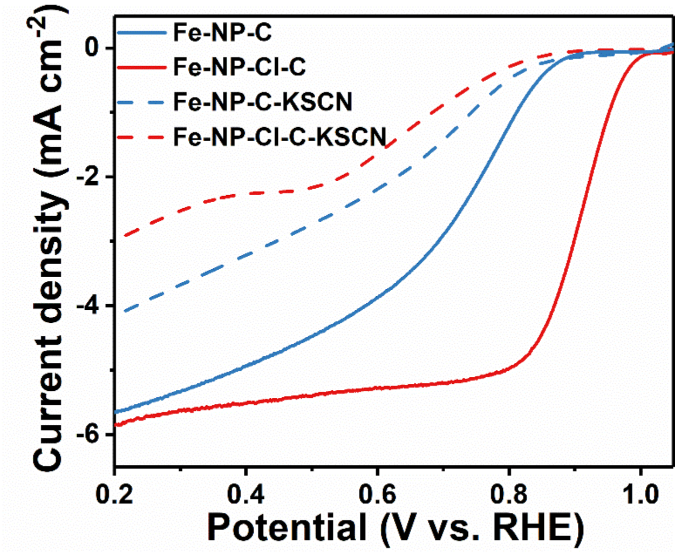

Figure S26. LSV curves of Fe-NP-C, Fe-NP-C-KSCN, Fe-NP-Cl-C and Fe-NP-Cl-C-KSCN in O_2-saturated 0.1 M KOH solution at a rotating speed of 1600 rpm.

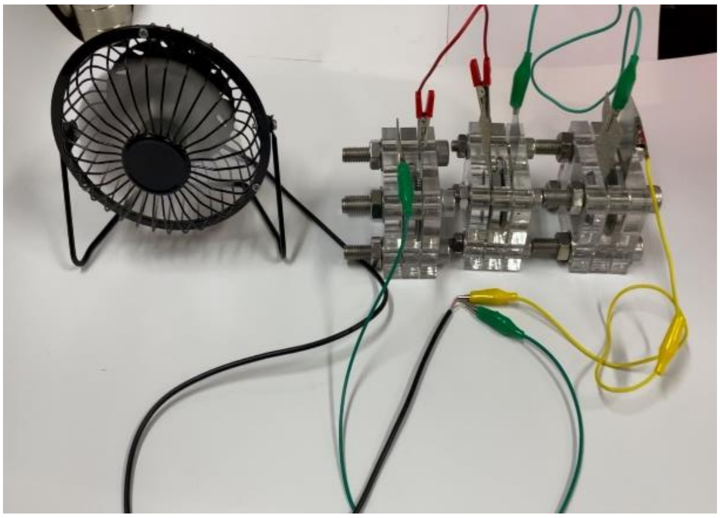

Figure S27. Photograph of electric fan powered by three Fe-NP-Cl-C-based Zn-air batteries in series.

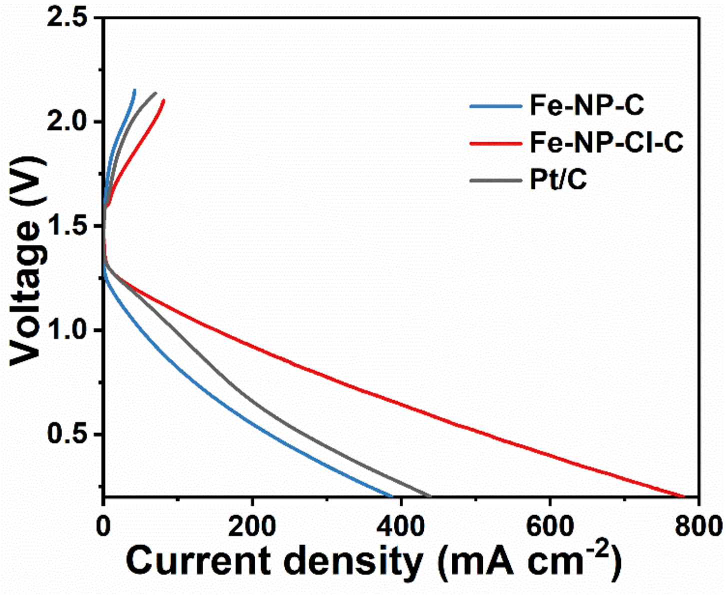

Figure S28. Charge-discharge curves of Fe-NP-C, Fe-NP-Cl-C and Pt/C catalysts.

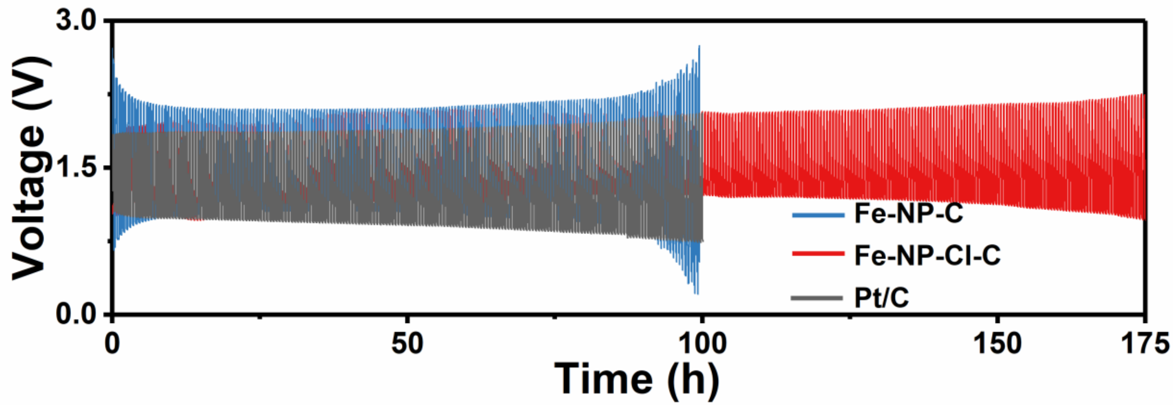

Figure S29. Long-term cycling performance of the Zn-air batteries based on Fe-NP-C, Fe-NP-Cl-C and Pt/C at the current density of 10 mA cm^{-2}.

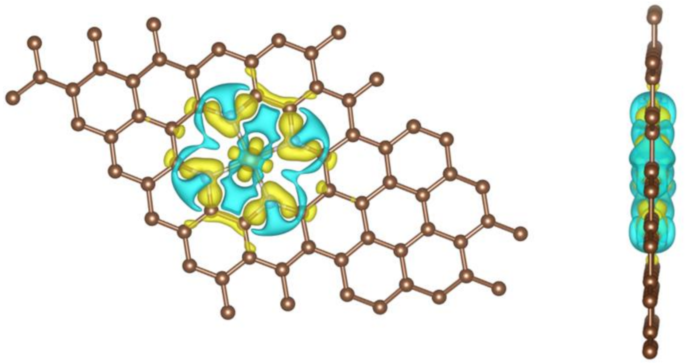

Figure S30. DFT calculation analyzes the charge density difference of Fe-N_4 between dopants and graphitic carbon layer. Electron accumulation is in blue and depletion in yellow.

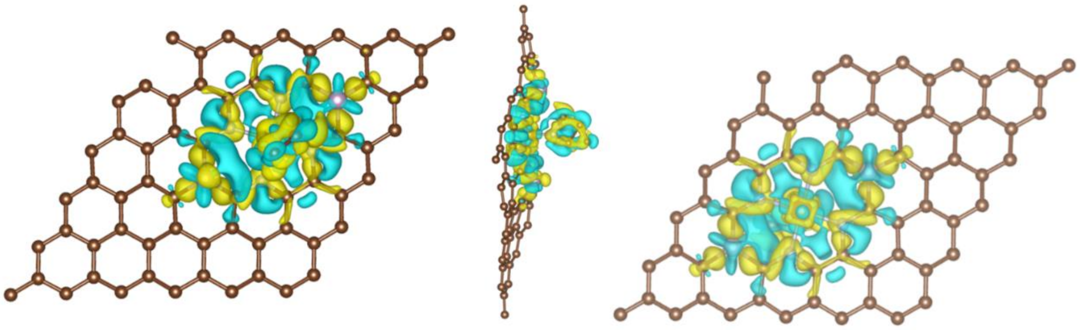

Figure S31. DFT calculation analyzes the charge density difference of P-Fe-N_5 between dopants and graphitic carbon layer. Electron accumulation is in blue and depletion in yellow.

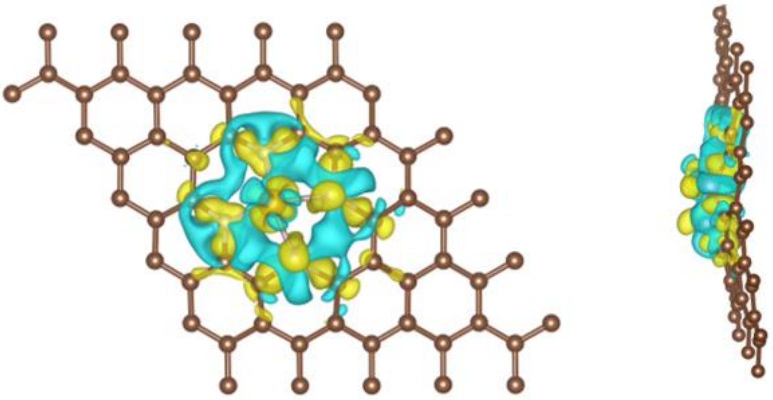

Figure S32. DFT calculation analyzes the charge density difference of Fe-N_2P_2 between dopants and graphitic carbon layer. Electron accumulation is in blue and depletion in yellow.

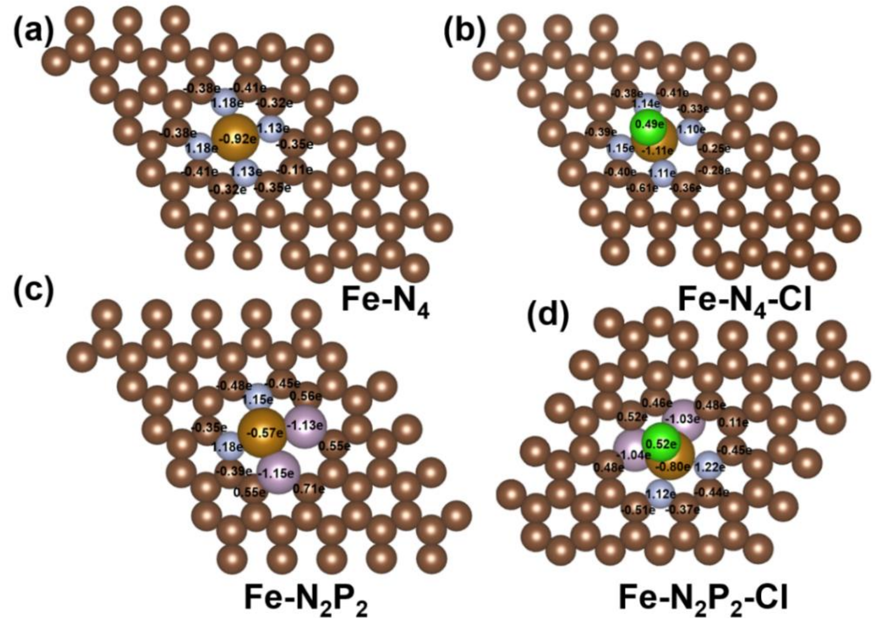

Figure S33. Bader charge analysis of (a) Fe-N_4, (b) Fe-N_4-Cl, (c) Fe-N_2P_2, and (d) Fe-N_2P_2-Cl.

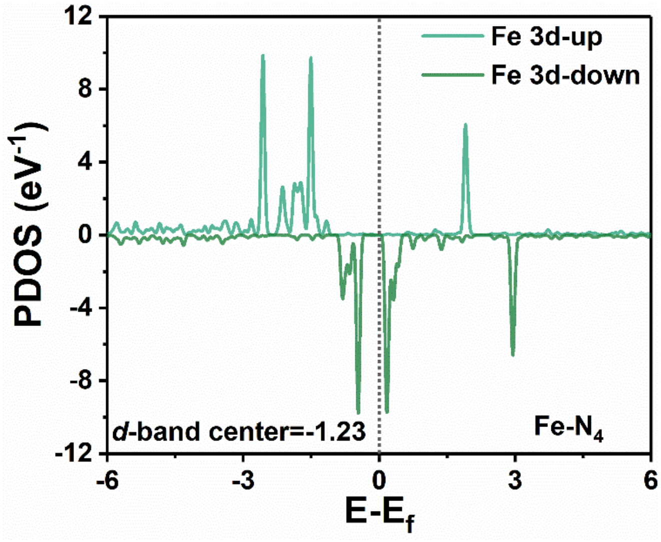

Figure S34. PDOS of Fe-N_4.

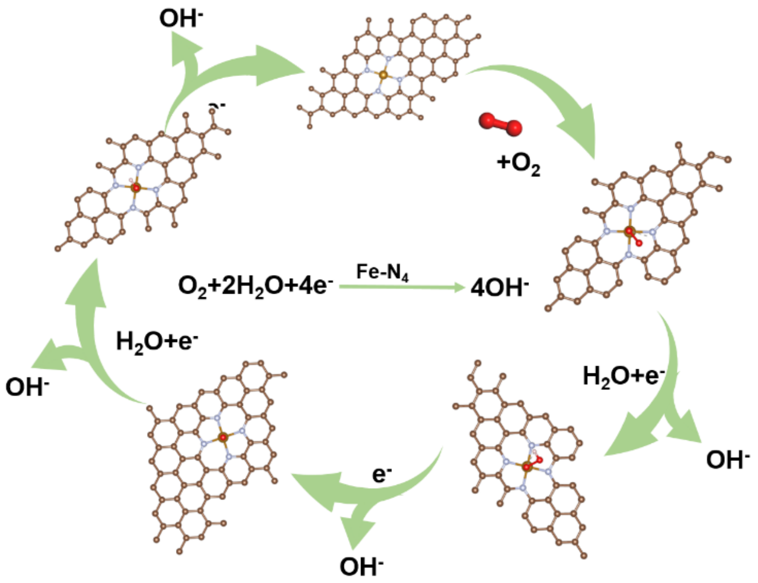

Figure S35. Illustration of the ORR reaction mechanism over Fe-N_4.

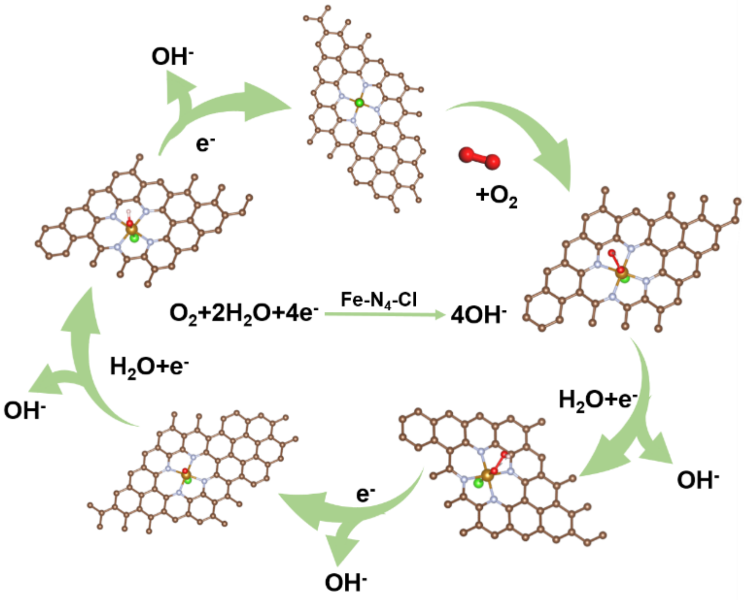

Figure S36. Illustration of the ORR reaction mechanism over Fe-N_4-Cl.

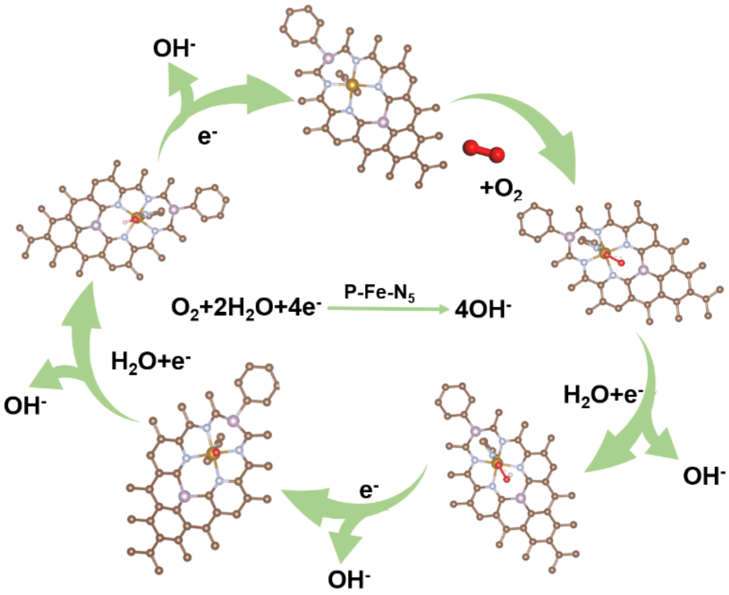

Figure S37. Illustration of the ORR reaction mechanism over P-Fe-N_5.

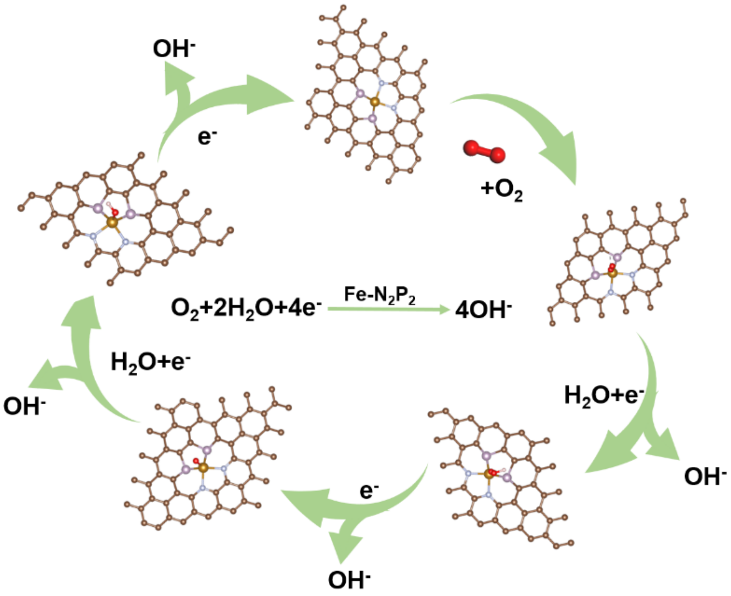

Figure S38. Illustration of the ORR reaction mechanism over Fe-N_2P_2.

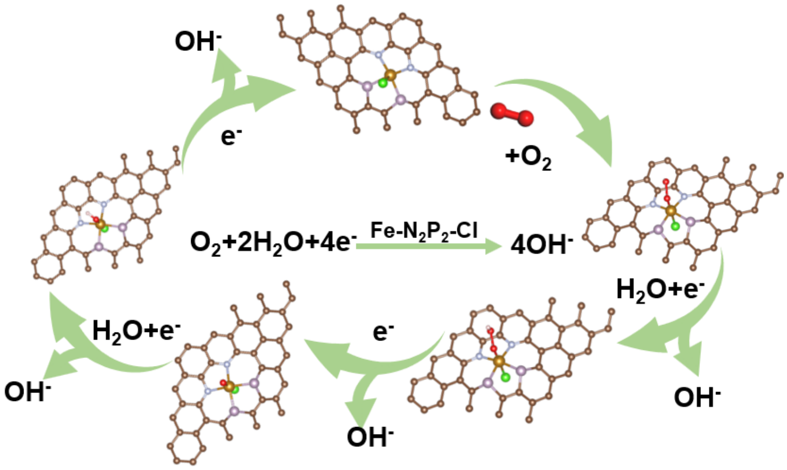

Figure S39. Illustration of the ORR reaction mechanism over Fe-N_2P_2-Cl.

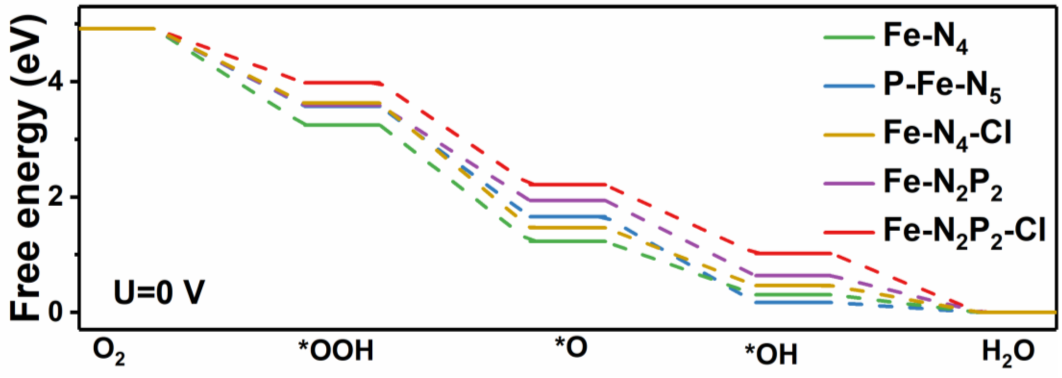

Figure S40. Free energy diagrams of ORR on Fe-N_4, Fe-N_4-Cl, P-Fe-N_5, Fe-N_2P_2 and Fe-N_2P_2-Cl models in alkaline media at U = 0 V.

Table S1. The pH variation of solutions.

	None	NaCl	KCl	$MgCl_2$	NaBr	$NaNO_3$
PA	1.15	-0.07	0.53	0.11	0.03	0.46
PA-Fe	0.45	-0.39	0.21	-0.24	-0.32	0.06

Table S2. The content of Na, Fe and P in PA-Fe, SE-PA-Fe, PA-Fe-OPD and NaCl-PA-Fe-OPD determined by ICP-AES.

Elements	Na (wt.%)	Fe (wt.%)	P (wt.%)
PA-Fe	0.02	19.9	14.1
SE-PA-Fe	0.36	17.4	13.8
PA-Fe-OPD	0.10	14.7	15.4
SE-PA-Fe-OPD	0.13	13.5	14.7

Table S3. The content of C, N, O, P, Fe and Cl loading in Fe-NP-C and Fe-NP-Cl-C tested by XPS.

Elements	C (at.%)	N (at.%)	O (at.%)	P (at.%)	Fe (at.%)	Cl (at.%)
Fe-NP-C	68.1	8.2	18.1	4.9	0.7	-
Fe-NP-Cl-C	85.6	3.4	8.2	1.4	0.7	0.7

Table S4. The content of Fe and P in Fe-NP-C and Fe-NP-Cl-C determined by ICP-AES.

Elements	Fe (wt.%)	P (wt.%)	Na (wt.%)
Fe-NP-C	3.9	6.4	-
Fe-NP-Cl-C	0.9	1.3	0.04

Table S5. The content of C, N, O, P, Fe and Cl loading in Fe-NP-C, Fe-NP-Cl-C, Fe-NP-Cl-C-KCl, Fe-NP-Br-C-NaBr and Fe-NP-C-$NaNO_3$ determined by XPS.

Elements	C (at.%)	N (at.%)	O (at.%)	P (at.%)	Fe (at.%)	Cl (at.%)	Br (at.%)
Fe-NP-C	68.1	8.2	18.1	4.9	0.7	-	-
Fe-NP-Cl-C	85.6	3.4	8.2	1.4	0.7	0.7	-
Fe-NP-Cl-C-KCl	82.8	3.6	11.1	1.1	0.6	0.8	-

Fe-NP-Br-C-NaBr	68.7	2.8	19.1	5.9	3.3	-	0.2
Fe-NP-C-$NaNO_3$	80.6	3.2	14.3	1.1	0.8	-	-

Table S6. Structural parameters of samples obtained by fitting the EXAFS data. There are the average coordination number (N), path distance (R), Debye-Waller factor (σ^2), threshold energy correction (ΔE), and the R-Factor of the fitting.

Sample	Path	CN	R(Å)	σ^2 (×10^{-3} $Å^2$)	ΔE_0 (eV)	R factor
Fe-NP-C	Fe-N	5.3	2.02	4.7	-1.92	0.016
	Fe-N	2.3	2.07	11.4		
Fe-NP-Cl-C	Fe-P	2.0	2.40	6.6	1.6	0.018
	Fe-Cl	0.6	2.20	11.2		

Table S7. Comparison of the ORR performance of Fe-NP-Cl-C with recent reported Fe/Co-based catalysts.

Samples	E_{onset} (V vs. RHE)	$E_{1/2}$ (V vs. RHE)	References
Fe-N/C-SAC	0.95	0.91	[2]
$FeCl_1N_4$/CNS	0.96	0.92	[3]
FeCo-N-HCN	0.98	0.86	[4]
O-Co-N/C	0.89	0.85	[5]
P/Fe-N-C	0.98	0.9	[6]
Fe-N/P-C	0.94	0.87	[7]
NiCo DASs/N-C	0.92	0.88	[8]
FeCoNC/SL	0.94	0.87	[9]
FeN_4-Ten	0.922	0.867	[10]
Fe_1Se_1-NC	1.00	0.88	[11]
Fe(Fe)-N/S-C	0.991	0.872	[12]
**Fe-NP-Cl-C**	**0.99**	**0.92**	**This work**

Table S8. Comparison of the Fe-NP-Cl-C-based Zn-air battery performance with recent reported Fe/Co-based catalysts.

Samples	Open Circuit Voltage (V)	Peak Power density ($mW\ cm^{-2}$)	References
Fe-N/P-C	1.42	133.2	[7]
FeCoN/SL	1.438	224.8	[9]
FeN_4-Ten	1.53	183	[10]
Fe-N-C	1.49	157	[13]
FeN_4Cl_1/NC	1.47	170	[14]
Co_1-N_3PS-HC	1.47	176	[15]
Fe-N@Ni-HCFs	1.51	172.2	[16]
FeN_4-O-NCR	1.47	214	[17]
Fe_3Co_7-NC	1.51	133	[18]
Fe-N/S-C	-	203	[19]
FeNC-TA	1.52	236	[20]
Fe, Cu DAs-NC	-	164	[21]

Fe SAC-MIL101-1000	-	192.3	[22]
FePc-NHCS-500	1.524	230	[23]
**Fe-NP-Cl-C**	**1.496**	**260**	**This work**

References

[1] a) F. G.KresseaJ, *Comp. Mater. Sci.* **1996**, *6*, 15-50; b) D. J. G. Kresse, *Phys. Rev. B* **1999**, *59*, 1758-1775.

[2] C. Xin, W. Shang, J. Hu, C. Zhu, J. Guo, J. Zhang, H. Dong, W. Liu, Y. Shi, *Adv. Funct. Mater.* **2021**, *32*, 2108345.

[3] Y. Han, Y. Wang, R. Xu, W. Chen, L. Zheng, A. Han, Y. Zhu, J. Zhang, H. Zhang, J. Luo, C. Chen, Q. Peng, D. Wang, Y. Li, *Energ. Environ. Sci.* **2018**, *11*, 2348-2352.

[4] H. Li, Y. Wen, M. Jiang, Y. Yao, H. Zhou, Z. Huang, J. Li, S. Jiao, Y. Kuang, S. Luo, *Adv. Funct. Mater* **2021**, *31,* 2011289.

[5] W. Zhang, C. H. Xu, H. Zheng, R. Li, K. Zhou, *Adv. Funct. Mater.* **2022**, 2200763.

[6] Y. Zhou, R. Lu, X. Tao, Z. Qiu, G. Chen, J. Yang, Y. Zhao, X. Feng, K. Mullen, *J. Am. Chem. Soc.* **2023**, *145*, 3647-3655.

[7] K. Yuan, D. Lutzenkirchen-Hecht, L. Li, L. Shuai, Y. Li, R. Cao, M. Qiu, X. Zhuang, M. K. H. Leung, Y. Chen, U. Scherf, *J. Am. Chem. Soc.* **2020**, *142*, 2404-2412.

[8] M. Li, H. Zhu, Q. Yuan, T. Li, M. Wang, P. Zhang, Y. Zhao, D. Qin, W. Guo, B. Liu, X. Yang, Y. Liu, Y. Pan, *Adv. Funct. Mater.* **2022**, *33,* 2210867.

[9] X. Zhao, J. Chen, Z. Bi, S. Chen, L. Feng, X. Zhou, H. Zhang, Y. Zhou, T. Wagberg, G. Hu, *Adv. Sci.* **2023**, *10*, 2205889.

[10] B. F. Ji, J. L. Gou, Y. P. Zheng, X. L. Zhou, P. Kidkhunthod, Y. H. Wang, Q. Y. Tang, Y. B. Tang, *Adv. Mater.* **2022**, *34,* 2202714.

[11] Z. Chen, X. Su, J. Ding, N. Yang, W. Zuo, Q. He, Z. Wei, Q. Zhang, J. Huang, Y. Zhai, *Appl. Catal. B-Environ* **2022**, *308,* 121206.

[12] X. Li, X. Yang, L. Liu, H. Zhao, Y. Li, H. Zhu, Y. Chen, S. Guo, Y. Liu, Q. Tan, G. Wu, *Acs Catal.* **2021**, *11*, 7450-7459.

[13] L. Deng, L. Qiu, R. Hu, L. Yao, Z. Zheng, X. Ren, Y. Li, C. He, *Appl. Catal. B-Environ*

2022, *305,* 121058.

[14] L. Hu, C. Dai, L. Chen, Y. Zhu, Y. Hao, Q. Zhang, L. Gu, X. Feng, S. Yuan, L. Wang, B. Wang, *Angew. Chem., Int. Ed. Engl.* **2021**, *60*, 27324-27329.

[15] Y. Chen, R. Gao, S. Ji, H. Li, K. Tang, P. Jiang, H. Hu, Z. Zhang, H. Hao, Q. Qu, X. Liang, W. Chen, J. Dong, D. Wang, Y. Li, *Angew. Chem., Int. Ed. Engl.* **2021**, *60*, 3212-3221.

[16] Y. H. Tian, Z. Z. Wu, M. Li, Q. Sun, H. Chen, D. Yuan, D. J. Deng, B. Johannessen, Y. Wang, Y. L. Zhong, L. Xu, J. Lu, S. Q. Zhang, *Adv. Funct. Mater* **2022**, *32,* 2209273.

[17] L. B. Zong, K. C. Fan, P. Li, F. H. Lu, B. Li, L. Wang, *Adv. Energy. Mater.* **2023**, 13, 2203611.

[18] T. T. Gu, D. T. Zhang, Y. Yang, C. Peng, D. F. Xue, C. Y. Zhi, M. Zhu, J. Liu, *Adv. Funct. Mater.* **2022**, 2212299.

[19] L. Li, S. Huang, R. Cao, K. Yuan, C. Lu, B. Huang, X. Tang, T. Hu, X. Zhuang, Y. Chen, *Small* **2022**, *18*, 2105387.

[20] X. Ao, Y. Ding, G. Nam, L. Soule, P. Jing, B. Zhao, J. Y. Hwang, J. H. Jang, C. Wang, M. Liu, *Small* **2022**, *18*, 2203326..

[21] F. Kong, M. Wang, Y. Huang, G. Meng, M. Chen, H. Tian, Y. Chen, C. Chen, Z. Chang, X. Cui, J. Shi, *Energy Storage Mater.* **2023**, *54*, 533-542.

[22] X. Xie, L. Peng, H. Yang, G. I. N. Waterhouse, L. Shang, T. Zhang, *Adv. Mate.r* **2021**, *33*, 2101038.

[23] H. Zhang, Z. Zhang, Z. Zhang, Y. Li, Y. Hou, P. Liu, B. Xu, H. Zhang, Y. Liu, J. Guo, *Chem. Eng. J.* **2023**, *469*, 143996.
